# graph-GPA: A graphical model for prioritizing GWAS results and investigating pleiotropic architecture

**DOI:** 10.1371/journal.pcbi.1005388

**Published:** 2017-02-17

**Authors:** Dongjun Chung, Hang J. Kim, Hongyu Zhao

**Affiliations:** 1 Department of Public Health Sciences, Medical University of South Carolina, Charleston, South Carolina, United States of America; 2 Department of Mathematical Sciences, University of Cincinnati, Cincinnati, Ohio, United States of America; 3 Department of Biostatistics, Yale School of Public Health, New Haven, Connecticut, United States of America; 4 Program in Computational Biology and Bioinformatics, Yale University, New Haven, Connecticut, United States of America; 5 Department of Genetics, Yale School of Medicine, New Haven, Connecticut, United States of America; 6 VA Cooperative Studies Program Coordinating Center, West Haven, Connecticut, United States of America; Johns Hopkins University, UNITED STATES

## Abstract

Genome-wide association studies (GWAS) have identified tens of thousands of genetic variants associated with hundreds of phenotypes and diseases, which have provided clinical and medical benefits to patients with novel biomarkers and therapeutic targets. However, identification of risk variants associated with complex diseases remains challenging as they are often affected by many genetic variants with small or moderate effects. There has been accumulating evidence suggesting that different complex traits share common risk basis, namely pleiotropy. Recently, several statistical methods have been developed to improve statistical power to identify risk variants for complex traits through a joint analysis of multiple GWAS datasets by leveraging pleiotropy. While these methods were shown to improve statistical power for association mapping compared to separate analyses, they are still limited in the number of phenotypes that can be integrated. In order to address this challenge, in this paper, we propose a novel statistical framework, graph-GPA, to integrate a large number of GWAS datasets for multiple phenotypes using a hidden Markov random field approach. Application of graph-GPA to a joint analysis of GWAS datasets for 12 phenotypes shows that graph-GPA improves statistical power to identify risk variants compared to statistical methods based on smaller number of GWAS datasets. In addition, graph-GPA also promotes better understanding of genetic mechanisms shared among phenotypes, which can potentially be useful for the development of improved diagnosis and therapeutics. The R implementation of graph-GPA is currently available at https://dongjunchung.github.io/GGPA/.

## Author summary

Recently, there has been accumulating evidence suggesting pleiotropy, i.e., genetic components shared across multiple phenotypes. Incorporation of pleiotropy in genetic analysis might improve statistical power to identify risk associated genetic variants. Several statistical approaches have been proposed to utilize pleiotropy for association mapping but they are currently still limited to a relatively small number of phenotypes, e.g., a pair of phenotypes. This restricts potential gain in statistical power in association mapping and investigation of pleiotropic structure among a large number of phenotypes. In order to address this challenge, in this paper, we propose graph-GPA, a novel statistical framework to integrate a large number of phenotypes using a hidden Markov random field architecture. Application of the proposed statistical method to GWAS datasets for 12 phenotypes showed that graph-GPA does not only provide a parsimonious representation of genetic relationship among these phenotypes, but also identify significantly larger number of novel genetic variants that are potentially functional. We believe that this novel approach might help investigation of common etiology and improvement of diagnosis and therapeutics.

## Introduction

Genome-wide association studies (GWAS) have identified many single-nucleotide polymorphisms (SNPs) associated with various phenotypes, including cancer, diabetes, and autoimmune diseases, among others. As of July 2016, more than 20,000 SNPs have been reported to be significantly associated with at least one complex trait in the NHGRI-EBI catalog of published GWAS [1] (http://www.ebi.ac.uk/gwas/). Despite these great achievements, researchers have had concerns about the fact that these significantly associated SNPs could explain only a small portion of genetic contributions to complex traits/diseases, which is referred to as *“missing heritability”* [2–4]. For example, while human height is known to be highly heritable and its heritability is estimated to be up to 80% [5], genome-wide significant SNPs (*p*-value < 5 × 10^−8^ after Bonferroni correction) together can only explain 16% of variation in height [6]. Researchers have tried to identify sources of this missing heritability phenomenon and progress has been made towards explaining some sources of the phenomenon. Among these efforts, Yang et al. [7] reported that, in their cohort genotyped on HumanCNV370-Quad v3.0 BeadChips (∼351K SNPs) or Human610-Quad v1.0 BeadChips (∼582K SNPs) platforms, all genotyped common SNPs together can explain 45% of variation in human height using a random effects model. This result suggests that a large proportion of the heritability is not actually missing: given the limited sample size, many individual effects of genetic markers are too weak to pass the genome-wide significance, and thus those variants remain undiscovered, which is usually referred to as “polygenicity”.

Although polygenicity provides attractive explanation for the missing heritability phenomenon, the polygenic architecture imposes a great practical challenge in GWAS. While identification of genetic variants with small effect sizes requires a larger sample, it is often not a practical option to recruit a larger sample because recruitment may be expensive and time-consuming. Hence, it would be desirable if we could increase statistical power to detect risk associated genetic variants with smaller effect sizes, without extensive additional subject recruitment. Integrative analysis of genetic and genomic data has recently been considered as a promising direction, including combining GWAS data of multiple genetically related phenotypes. In the last few years, researchers have provided convincing evidence of “pleiotropy”, i.e. the sharing of genetic factors, especially between human complex traits. For example, a systematic analysis of the NHGRI GWAS Catalog demonstrated that 16.9% of the reported genes and 4.6% of the reported SNPs are associated with multiple traits [8]. As a specific example, recent genetic studies for five psychiatric disorders reported high genetic correlation between schizophrenia and bipolar disorders [9, 10]. Furthermore, a series of studies have shown that integration of genetic data for multiple phenotypes can boost statistical power to identify risk associated genetic variants [11–15]. For example, Andreassen et al. [12] showed that exploiting the pleiotropy between schizophrenia and cardiovascular disease greatly improved the statistical power to detect schizophrenia-associated genetic variants.

In order to address these challenges and opportunities, Chung and others proposed a statistical framework, namely GPA (Genetic analysis incorporating Pleiotropy and Annotation), that integrates genetic data for multiple phenotypes [11]. Using extensive simulation studies, the authors showed that GPA outperforms other methods utilizing pleiotropy for association mapping, such as conditional FDR approach [13]. The GPA algorithm was successfully applied to a joint analysis of five psychiatric disorders, including attention deficit hyperactivity disorder (ADHD), autism spectrum disorder (ASD), bipolar disorder (BPD), major depressive disorder (MDD), and schizophrenia (SCZ). For example, GPA detected strong genetic relationship between BPD and SCZ and the joint analysis of GWAS data for these two diseases significantly improved statistical power to identify genetic variants associated with risks of BPD and SCZ. Specifically, at the local false discovery rate (FDR) of 0.05, separate analyses identified only 14 and 409 genetic variants associated with BPD and SCZ, respectively, while joint analysis using GPA uncovered 383 and 821 associated genetic variants. Furthermore, some of these additionally identified SNPs were confirmed by the findings of Cross-Disorder Group of the Psychiatric Genomics Consortium [9, 10].

This paper is motivated by the joint analysis of GWAS data for 12 phenotypes, including psychiatric disorders, autoimmune diseases, lipid-related phenotypes, and cardiovascular phenotypes. The extensive integrative analysis is of interest because it is expected that genetic basis is not shared only within a phenotype group (e.g., psychiatric disorders) but also between phenotype groups (e.g., between psychiatric disorders and autoimmune diseases), as suggested in recent literature [16]. However, the state-of-the-art approaches to integrating GWAS datasets are still limited to a relatively small number of phenotypes, e.g., a pair of phenotypes [11–15, 17]. For example, GPA considers all possible combinations of phenotype associations of which the number increases exponentially with the number of phenotypes. Specifically, the joint analysis of 12 phenotypes requires consideration of 4,096 combinations of association status, which is computationally prohibitive and can generate various estimation issues. As a result, information sharing is permissible only among a small number of phenotypes with existing methods, limiting potential improvement in statistical power to identify risk associated genetic variants. This limitation highlights the urgent need for a statistical framework that enables integration across a large number of GWAS datasets.

In order to address the challenge, in this paper, we propose a novel statistical framework, namely graph-GPA. Specifically, we utilize the Markov random field (MRF) architecture to model hidden binary indicators for association of a SNP with each phenotype. The MRF architecture does not only provide a parsimonious representation of genetic relationship among phenotypes, but also improves statistical power to identify risk associated genetic variants by sharing information across GWAS data for these phenotypes. This paper is structured as follows. We first propose our novel Bayesian hierarchical model for the joint analysis of GWAS data for multiple phenotypes. Then, we evaluate the proposed method with simulation studies and conduct the joint analysis of GWAS datasets for 12 phenotypes, including psychiatric disorders, autoimmune diseases, lipid-related phenotypes, and cardiovascular phenotypes. Our real data analysis reveals genetic mechanisms shared among the phenotypes. Furthermore, graph-GPA improves statistical power to identify both risk variants associated with each phenotype (bipolar disorders) and those with pleiotropic effects (psychiatric disorders and inflammatory bowel diseases).

## Materials and methods

### graph-GPA model

In this paper, we integrate GWAS data for multiple phenotypes at the level of summary statistics rather than full genotype and phenotype datasets. For example, they might be *p*-values obtained from a logistic regression of phenotype on genotype or a contingency table for genotype-phenotype association. This approach allows broader application of the proposed method because summary statistics are more readily available. Let *p*_*it*_ denote the *p*-value of association testing of SNP *t* = 1, 2, ⋯, *T* with phenotype *i* = 1, 2, ⋯, *n*. To facilitate data visualization and modeling, we transformed *p*_*it*_ as *y*_*it*_ = Φ^−1^(1 − *p*_*it*_), where Φ(⋅) is the cumulative distribution of the standard normal variable [18]. Let *e*_*it*_ denote the latent (unobservable) association indicator for *t*-th SNP and *i*-th phenotype, where *e*_*it*_ = 1 if SNP *t* is associated with phenotype *i* and *e*_*it*_ = 0 otherwise. We model the density of *y*_*it*_ given the latent association status *e*_*it*_ by a normal mixture

$$p\left(y_{it}|e_{it},\mu_{i},\sigma_{i}^{2}\right)=e_{it}\text{LN}\left(y_{it};\mu_{i},\sigma_{i}^{2}\right)+\left(1-e_{it}\right)\text{N}\left(y_{it};0,1\right),$$ (1)

where LN(*y*; *μ*, *σ*^2^) and *N*(*y*; 0, 1) denote the log-normal density with mean $e^{\mu +\sigma^{2}/2}$ and the standard normal density, respectively. Here, the standard normal distribution assumption for background SNPs (*e*_*it*_ = 0) is equivalent to the theoretical null distribution assumption (uniformity of *p*-values) [18]. We assume the log-normal distribution for *y*_*it*_ corresponding to associated SNPs (*e*_*it*_ = 1) because it is likely that associated SNPs will only account for a negligible proportion of the SNPs with *p*-values larger than 0.5, i.e., *y*_*it*_ smaller than zero. We evaluated the appropriateness of these assumptions using real GWAS datasets and confirmed that no significant violation of these assumptions is detected (Sections 6 and 7 in S1 Text). However, we note that other distributions can also be considered, especially for associated SNPs, because we chose the log-normal distribution mainly due to convenience and interpretability. In order to check this issue and further confirm the robustness of our results, we also considered Gamma distribution as an alternative distribution for associated SNPs instead of log-normal distribution. The results indicate that our results are still robust to this alternative choice of emission distribution for associated SNPs (Section 8 in S1 Text).

For effective integration of multiple GWAS datasets for genetically related phenotypes, we suggest a graphical model based on an MRF [19] that represents a conditional independent structure for genetic relationship among phenotypes. Let *G* = (*V*,*E*) denote an MRF graph, where *V* = (*v*_1_, …, *v*_*n*_) are the vertices and *E* represents the edges such that *E*(*i*, *j*) = 1 if there is an edge between *v*_*i*_ and *v*_*j*_ and *E*(*i*, *j*) = 0 otherwise. Here, *v*_*i*_ corresponds to phenotype *i* and *E*(*i*, *j*) = 1 implies that phenotypes *i* and *j* are genetically correlated in the sense of conditional dependence. Assuming an auto-logistic spatial scheme, the conditional distribution of ***e***_*t*_ = (*e*_1*t*_, …, *e_nt_*) is written by

$$p\left(e_{t}|\alpha ,\beta ,G\right)=\text{C}\left(\alpha ,\beta ,G\right)\cdot \exp\left(\sum_{i=1}^{n}\alpha_{i}e_{it}+\sum_{i∼j}\beta_{ij}e_{it}e_{jt}\right),$$ (2)

where

$$\text{C}{\left(\alpha ,\beta ,G\right)}^{-1}=\sum_{e^{*}\in E}\exp\left(\sum_{i=1}^{n}\alpha_{i}e_{i}^{*}+\sum_{i∼j}\beta_{ij}e_{i}^{*}e_{j}^{*}\right),$$ (3)

E is the set of all possible values of $e^{*}=\left(e_{1}^{*},\ldots ,e_{n}^{*}\right)$, *β*_*ij*_ is the MRF coefficient for the pair of phenotypes *i* and *j*, and *i* ∼ *j* denotes that *v*_*i*_ is adjacent to *v*_*j*_, i.e., the sum of the second term is over all pairs of phenotypes *i* and *j* such that *E*(*i*, *j*) = 1.

For Bayesian inference, we introduce conjugate prior distributions for Model (1),

μi∼N(θμ,τμ2),BBσi2∼IG(aσ,bσ), (4)

where IG denotes the inverse-gamma distribution. For coefficients in the MRF Model (2), prior distributions are assumed as

αi∼N(θα,τα2),BBβij∼E(i,j)Γ(βij;aβ,bβ)+{1-E(i,j)}δ0(βij), (5)

where Γ(*a*, *b*) denotes the gamma distribution with mean *a*/*b* and *δ*_0_ denotes the Dirac delta function at zero. This prior setting for *β*_*ij*_ reflects the graph structure **G** by putting *β*_*ij*_ = 0 if there is no edge between phenotypes *i* and *j* in the graph, i.e., *E*(*i*, *j*) = 0. We put *p*(***G***) = 1/|*E*|, where |*E*| denotes the number of edges in the current graph ***G***, which leads the model to favor simple graphical representation with a small number of edges, i.e., the small number of *β*_*ij*_ having non-zero values. As informed by a referee, our estimation of the graphical structure **G** is conceptually similar to “learning the structure” of MRF in the machine learning literature. Specifically, in the “learning the structure” approach, the *L*_1_ (Lasso) regularization is directly imposed instead of using the prior distribution of **G** in our current model. This approach tends to lead to sparse models where many *β*_*ij*_ have zero values while not much sacrificing the model fit (e.g., [20–22]). In our simulation and application studies, weakly informative priors are used by setting the following hyperparameters: *θ*_*μ*_ = 0, $\tau_{\mu }^{2}=10000$, *θ*_*α*_ = 0, and $\tau_{\alpha }^{2}=10000$. We set *a*_*σ*_ = *b*_*σ*_ = 0.5 to put substantial prior mass on modest-sized variances. We put *a*_*β*_ = 4 and *b*_*β*_ = 2 to let most of *β*_*ij*_ with *E*(*i*, *j*) = 1 *a priori* distinct from zero, having mean 2 and variance 1, while allowing a handful of values be close to zero. Note that we defer the discussion about model robustness to the hyperparameters to the Results Section.

### Posterior inference

Given the *p*-values from GWAS datasets, we estimate the parameters of the graph-GPA model and other related interesting quantities based on posterior samples from a Markov Chain Monte Carlo (MCMC) sampler. Specifically, we implement a Metropolis-Hastings within Gibbs algorithm whose full details are provided in Section 1 in S1 Text. Here, we address a few key points about the MCMC implementation. As adopting some conjugate forms of prior distributions, most MCMC steps consist of Gibbs updating which is computationally efficient. To deal with the dimensional changing structure of *β*_*ij*_ given ***G***, we adopt the reversible jump MCMC [23] to add or remove edges in the graph ***G*** during the MCMC run.

The graph structure of ***G*** representing genetic relationship among phenotypes can be summarized by *p*(*E*(*i*, *j*)|***Y***), interpreted as the posterior probability that two phenotypes *i* and *j* are genetically correlated with each other. In addition, the posterior summary of *β*_*ij*_ can be interpreted as a relative metric to gauge the degree of correlation between phenotypes *i* and *j*. Using these posterior summaries, we can come up with several strategies to make an inference about the pairwise relationship between phenotypes. A convenient and widely used approach is to set an arbitrary threshold for *p*(*E*(*i*, *j*)|***Y***) to declare phenotypes *i* and *j* to be correlated, for example, *p*(*E*(*i*, *j*)|***Y***) > 0.5 as in [24, 25]. However, we found that this approach gives suboptimal results in estimation of a graph structure because it does not exclude the case that two phenotypes are connected in the graph (*E*(*i*, *j*) = 1) but their corresponding MRF coefficient (*β*_*i*, *j*_) is close to zero. Hence, here we adopt an alternative approach to improving specificity in identifying correlated pairs of phenotypes using both *p*(*E*(*i*, *j*)|***Y***) and the posterior summary of *β*_*i*, *j*_. Specifically, we declare phenotypes *i* and *j* to be correlated when *p*(*E*(*i*, *j*)|***Y***) > 0.5 and *β*_*ij*_ is significantly different from zero, e.g., *p*(*β_ij_* > 0|***Y***) > 0.95.

Association mapping of a single SNP on a specific phenotype is inferred from *p*(*e*_*it*_ = 1|**Y**), i.e., the posterior probability that SNP *t* is associated with phenotype *i*. SNPs associated with both *i*-th and *j*-th phenotypes can be inferred based on *p*(*e*_*it*_ = 1, *e*_*jt*_ = 1|**Y**) and SNPs associated with more than two phenotypes can also be identified in similar ways. We use the *direct posterior probability approach* [26] to control global false discovery rates to determine associated SNPs. Specifically, given the graph-GPA model fitting, we first sort SNPs by their local false discovery rates from the smallest to the largest. For example, we can sort SNPs by their local false discovery rates for phenotype *i*, denoted as *f*_*it*_ ≡ 1 − *p*(*e*_*it*_ = 1|**Y**). Then, we increase the threshold for local false discovery rates, *κ*_*i*_, from zero to one until

$${\text{Fdr}}_{i}\equiv \frac{\sum_{t=1}^{T}f_{it}1\left\{f_{it}\leq \kappa_{i}\right\}}{\sum_{t=1}^{T}1\left\{f_{it}\leq \kappa_{i}\right\}}\leq \tau ,$$

where *τ* is the pre-determined bound of global false discovery rates, and 1{⋅} is the indicator function with value one if the statement is true and zero otherwise. Finally, we declare SNPs with corresponding *f*_*it*_ < *κ*_*i*_ to be associated with phenotype *i*. Similarly, SNPs shared between phenotypes *i* and *j* can be identified by using

$${\text{Fdr}}_{ij}\equiv \frac{\sum_{t=1}^{T}f_{ijt}1\left\{f_{ijt}\leq \kappa_{ij}\right\}}{\sum_{t=1}^{T}1\left\{f_{ijt}\leq \kappa_{ij}\right\}}\leq \tau ,$$

where *f*_*ijt*_ ≡ 1 − *p*(*e*_*it*_ = 1, *e*_*jt*_ = 1|**Y**).

## Results

### Simulation studies

**Fig 1:**
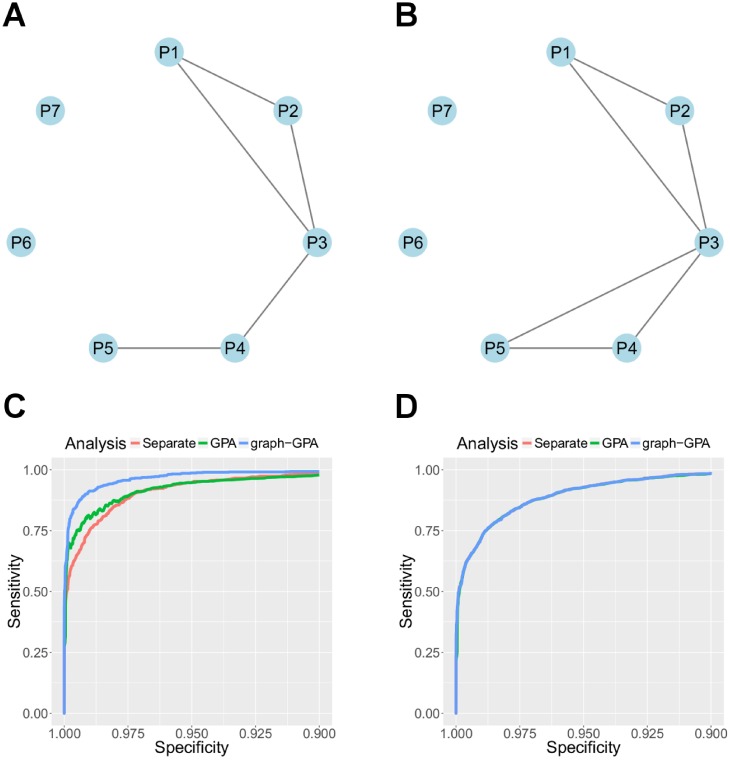
Simulation studies. (a) true phenotype graph, which was accurately recovered by graph-GPA. (b) phenotype graph estimated by GPA, which added an additional edge between P3 and P5. (c, d) receiver operating characteristic (ROC) curves for phenotypes P1 and P7. For a phenotype correlated with other phenotypes, such as P1 (c), joint analyses (graph-GPA and GPA) improve statistical power to identify associated SNPs compared to separate analyses, while graph-GPA further significantly outperforms GPA and separate analyses. In contrast, for independent phenotype such as P7 (d), joint and separate analyses have comparable statistical power in association mapping. Note that in (c) and (d), we show the averaged ROC for GPA, based on the pairs estimated to be correlated with P1 in (b), i.e., P1–P2 and P1–P3.

We conducted simulation studies to evaluate the performance of the proposed graph-GPA model. In the simulation studies, we mainly investigate the proposed model from the perspectives of 1) parameter estimation and false discovery rate control; 2) estimation of genetic relationship among diseases (graph structure); and 3) statistical power to identify risk-associated SNPs. We generated our simulation data as follows. First, we assumed the true phenotype graph (**G**) as depicted in Fig 1a. Specifically, we considered a group of tightly linked phenotypes (P1, P2, and P3) and a group of weakly linked phenotypes (P3, P4, and P5). In addition, we also assumed two isolated phenotypes (P6 and P7) as negative controls. Given this graph, we set *α*_1_ = −4.7, *α*_2_ = −3.0, *α*_3_ = −5.5, *α*_4_ = −4.8, *α*_5_ = −3.6, *α*_6_ = −2.5, and *α*_7_ = −3.5, while setting *β*_12_ = 4.0, *β*_13_ = 1.8, *β*_23_ = 2.3, *β*_34_ = 2.5, and *β*_45_ = 5.0 (all the remaining *β*_*ij*_ were set to zeros). These MRF coefficients were determined based on the estimated coefficients obtained using real GWAS datasets. Then, given the MRF coefficients, we generated association status of 20,000 common SNPs (*e*_*it*_) from the model in the Eq (2) by running the Gibbs sampler for 1,000 iterations [25]. Finally, given the association status of SNPs, we generated *y*_*it*_ from $N(\mu_{i},\sigma_{i}^{2})$ if *e*_*it*_ = 1, and *N*(0, 1) if *e*_*it*_ = 0, where ***μ*** = (1.1, 1.0, 1.2, 1.2, 1.3, 1.1, 1.3) and ***σ*** = (0.4, 0.3, 0.35, 0.3, 0.45, 0.4, 0.3). Note that we set the signal strengths relatively weak in order to mimic the distribution of *p*-values in real GWAS datasets. We applied the graph-GPA model with 10,000 burn-in and 40,000 main MCMC iterations to the simulation dataset. We also analyzed the simulation dataset with GPA as a representative method that can utilize a smaller number of phenotypes because GPA outperformed other currently available methods such as conditional FDR [12, 13] in previous studies [11].

We first evaluated the estimation accuracy of the proposed method. Figure A in S1 Text indicates that all the parameters are accurately estimated with high confidence. Specifically, for all parameters, the point estimates are close to true values and their 95% credible intervals always include true values. In addition, Figure B in S1 Text indicates that the global FDR is well controlled at the nominal FDR level for a wide range of FDR levels, regardless of whether it is for genetically related phenotypes or independent phenotypes. We then evaluated the estimation of genetic relationship among phenotypes. Fig 1a and 1b show the phenotype graph estimated using graph-GPA and GPA, respectively. We can see that graph-GPA accurately recovers true phenotype graph, while GPA adds an additional edge between P3 and P5. This is essentially because P3 and P5 are still correlated (which GPA infers) although they are conditionally independent given P4 (which graph-GPA infers). The result implies that graph-GPA has potential to better prioritize important pairs of genetically correlated phenotypes while it also provides more parsimonious representation of genetical correlation.

Finally, we evaluated the SNP prioritization performance of graph-GPA. As a baseline, we ran a null model without interaction term (*β*_*ij*_) as follows:

$$P\left(e_{t}|\alpha ,G\right)\propto \exp\left\{\sum_{i}\alpha_{i}e_{it}\right\}.$$ (6)

The no interaction model is interpreted as a special case of our suggested model in Eq (2) by putting the point mass at zero for prior distribution of *β*_*ij*_, i.e., the extremely strong prior belief that the phenotypes are independent. Note that these models correspond to separate analyses of GWAS datasets because they do not share information between phenotypes. Fig 1c shows the receiver operating characteristic (ROC) curves for graph-GPA, GPA, and separate analysis for the phenotype P1, which is genetically highly correlated with other phenotypes. Compared to the separate analysis, both graph-GPA and GPA improve area under the curve (AUC) values. More importantly, graph-GPA significantly outperforms GPA in the sense of AUC, due to more extensive information sharing across phenotypes. On the other hand, for independent phenotypes such as P7 illustrated in Fig 1d, there is no gain in AUC by employing graph-GPA or GPA compared to the separate analysis. The ROC curves for all the phenotypes are also provided in Section 5 of S1 Text and our conclusion remains valid across seven phenotypes. Tables A and C in S1 Text show numbers of SNPs identified by graph-GPA and in separate analyses at nominal FDR level of 10%. As expected, graph-GPA identified significantly larger number of associated SNPs (Table A in S1 Text) than separate analyses (Table C in S1 Text). In summary, graph-GPA significantly improves statistical power to identify associated SNPs compared to GPA and separate analyses by sharing information among larger number of phenotypes. Additionally, graph-GPA promotes understanding of genetical relationship among phenotypes by providing a parsimonious representation of pleiotropic architecture among these phenotypes.

### graph-GPA provides interpretable and stable representation of pleiotropic architecture

To evaluate the potential of the proposed model for genetic studies, we applied the proposed graph-GPA model and the GPA model to the GWAS data of European ancestry for 12 phenotypes, using summary statistics that are publicly available from consortium websites. Specifically, we considered 1) five psychiatric disorders, including attention deficit/hyperactivity disorder (ADHD), autism spectrum disorder (ASD), bipolar disorder (BPD), major depressive disorder (MDD), and schizophrenia (SCZ) from the Psychiatric Genomics Consortium [9, 10] (http://www.med.unc.edu/pgc); 2) three autoimmune diseases, including Crohn’s disease (CD) and ulcerative colitis (UC) from the International Inflammatory Bowel Disease Genetics Consortium [27, 28] (http://www.ibdgenetics.org/) and rheumatoid arthritis (RA) [29] (http://www.broadinstitute.org/ftp/pub/rheumatoid_arthritis/Stahl_etal_2010NG/); 3) two lipid-related phenotypes, including high-density lipoprotein (HDL) from the Global Lipids Consortium [30] (http://csg.sph.umich.edu//abecasis/public/lipids2010/) and type 2 diabetes (T2D) from the DIAbetes Genetics Replication And Meta-analysis Consortium [31] (http://diagram-consortium.org); and 4) two cardiovascular phenotypes, including coronary artery disease (CAD) from the CARDIoGRAM Consortium [32] (http://www.cardiogramplusc4d.org/data-downloads/) and systolic blood pressure (SBP) from the International Consortium for Blood Pressure [33] (http://www.georgehretlab.org/icbp_088023401234-9812599.html). We used the intersection of SNPs among these datasets, which consists of 228,944 SNPs. For the graph-GPA model, we collected the posterior sample of 40,000 MCMC iterations, removing the first 10,000 iterations as burn-in. The MCMC converged quickly to a stationary distribution (Figure K in S1 Text) and our sensitivity analysis results indicate that the model is not sensitive to misspecification of hyperparameters (Tables E and F in S1 Text).

**Fig 2:**
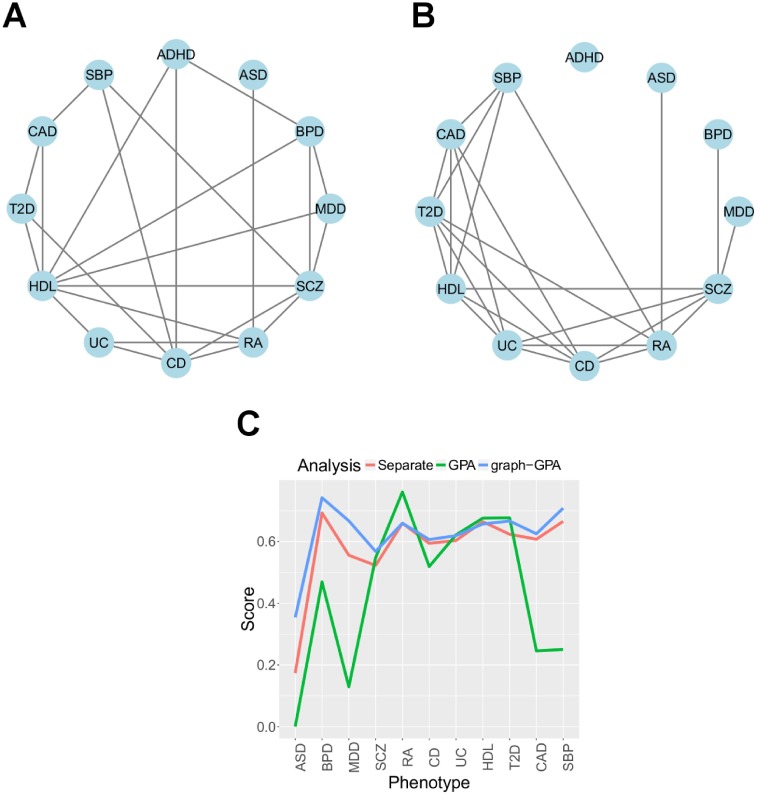
GWAS of twelve phenotypes. (a, b) phenotype graphs estimated by graph-GPA and GPA. graph-GPA provides more parsimonious representation of genetic relationship among phenotypes than GPA. (c) GenoCanyon scores for SNPs identified in separate analysis, by GPA, and by graph-GPA. GenoCanyon analysis shows that novel genetic variants identified by graph-GPA might be as functional as or more functional than those identified in separate analyses.

Fig 2a shows the phenotype graph estimated using the graph-GPA approach, where edges are connected if *p*(*E*(*i*, *j*)|***Y***) > 0.5 and the 95% credible interval of *β*_*ij*_ does not include zero. Tables G and H in S1 Text show estimates of *P*(*G*_*ij*_|**Y**) and *β*_*ij*_, which demonstrates the effectiveness of our approach in constructing a phenotype graph using both *β*_*ij*_ and *G*_*ij*_, discussed in the Materials and Methods Section. Specifically, by additionally considering credible interval of *β*_*ij*_, we could exclude the case to have nonzero *E*(*i*, *j*) with *β*_*ij*_ close to zero. In Fig 2a, as expected, clinically related phenotypes make a cluster, e.g., a psychiatric disorder cluster of ADHD-BPD-SCZ-MDD and an autoimmune disease cluster of CD-UC-RA. In addition, this phenotype graph indicates pleiotropy between psychiatric disorders and autoimmune diseases, also recently reported [34, 35]. On the other hand, the pleiotropy between T2D and CAD, and between cardiovascular complications and diabetes in general, has also been reported in multiple literature [36–38]. Fig 2b shows the phenotype graph estimated using the GPA algorithm. Although some key edges (such as RA-ASD) are still observed here, the phenotype graph estimated using GPA is denser than that estimated using graph-GPA and this makes it challenging to interpret genetic relationship among phenotypes. For example, all of T2D, CAD, UC, CD, and RA are tightly linked in the phenotype graph estimated using the GPA model. Moreover, in the graph, we also lost some edges between phenotypes, for example, ADHD is totally isolated from all the other phenotypes.

In order to further demonstrate stability of our findings, we introduced slight perturbation to the dataset. Specifically, we partitioned the cohorts for RA into two groups (WTCCC/EIRA/CANADA vs. NARAC-I/NARAC-III/BRASS, namely cohort groups A and B, respectively; see [29] for more details about these cohorts) and fitted graph-GPA models to each of two GWAS datasets (i.e., based on each RA GWAS data while GWAS data for all the remaining 11 phenotypes remain fixed). In general, the estimated phenotype graph for each RA cohort group (Figure L in S1 Text) is similar to the phenotype graph estimated using the full RA cohorts (Fig 2a). Not surprising, the estimated phenotype graph for each RA cohort group is slightly sparser than that using the full RA cohorts because we significantly reduce the sample size for RA by partitioning its cohorts into two groups. Although the weaker signals for RA affected the estimated graph, its effects are essentially local and limited to the first neighbors of RA in the estimated phenotype graphs. These results indicate that the proposed graph-GPA model can achieve a good balance between sensitivity and specificity in construction of a phenotype graph, which can be beneficial for prioritizing important pairs of phenotypes for further investigation of common etiology.

### graph-GPA allows robust identification of novel, potentially functional genetic variants

We next evaluated the association mapping performance of graph-GPA model. Table 1 shows the number of identified SNPs when we control the global FDR for each phenotype or each pair of phenotypes at the nominal level of 10%. These results confirm our finding in the previous section in the sense of identified SNPs. Specifically, we observe strong pleiotropy between BPD and SCZ, among autoimmune diseases (RA, CD, and UC), and between T2D and CAD, among others. It might look contradictory to observe that ADHD does not share SNPs with any other phenotypes in Table 1 while ADHD is linked to BPD, CD, and HDL in Fig 2a. However, this is likely due to less confidence in identification of SNPs associated with ADHD with weak effect sizes and, as a result, no SNP could pass the cut-off at the nominal FDR level. We confirmed this by observing that SNPs associated with ADHD were identified when we used looser FDR level (Tables M and N in S1 Text). In addition, as a baseline, Table 2 shows the number of SNPs identified using the null model (i.e., without interaction terms, corresponding to separate analysis) at the same global FDR level. As expected, in this case, a much lower number of SNPs are shared between phenotypes, which means that separate analysis has reduced statistical power in identifying pleiotropic SNPs. More importantly, the numbers of SNPs associated with each phenotype (diagonal terms in Table 2) were also smaller than those in Table 1. This implies that information sharing via the graph-GPA model significantly boosts statistical power to identify SNPs associated with each phenotype as well. Finally, as in the previous section, we further demonstrated stability of the results in Table 1 by partitioning the cohorts for RA into two groups, fitting graph-GPA models for each of two GWAS datasets, and evaluating their association mapping results (Tables I–L in S1 Text). As before, the association mapping results for each RA cohort group are similar to those obtained using the full RA cohorts. Also, such perturbation in dataset had local effects; for the pairs involving RA, less associated SNPs were identified as the sample size for RA decreased in this case. In summary, graph-GPA robustly improved statistical power to identify both SNPs with pleiotropic effects and SNPs associated with each phenotype by information sharing across wider range of phenotypes.

**Table 1:** GWAS of 12 phenotypes (graph-GPA analysis): Numbers of SNPs identified to be associated with each pair of phenotypes by controlling the global FDR at nominal level of 10%. Diagonal elements show the number of SNPs inferred to be associated with each phenotype when the global FDR is controlled at the same level.

	ADHD	ASD	BPD	MDD	SCZ	RA	CD	UC	HDL	T2D	CAD	SBP
ADHD	0	0	0	0	0	0	0	0	0	0	0	0
ASD	0	9	0	0	0	0	0	0	0	0	0	0
BPD	0	0	81	0	35	8	14	3	5	0	0	0
MDD	0	0	0	20	1	1	0	0	0	0	0	0
SCZ	0	0	35	1	415	72	63	48	44	3	18	17
RA	0	0	8	1	72	689	296	265	44	13	17	18
CD	0	0	14	0	63	296	2343	854	126	26	37	23
UC	0	0	3	0	48	265	854	1799	119	17	35	14
HDL	0	0	5	0	44	44	126	119	894	71	99	17
T2D	0	0	0	0	3	13	26	17	71	282	61	12
CAD	0	0	0	0	18	17	37	35	99	61	320	47
SBP	0	0	0	0	17	18	23	14	17	12	47	169

**Table 2:** GWAS of 12 phenotypes (separate analysis): Numbers of SNPs identified to be associated with each pair of phenotypes by controlling the global FDR at nominal level of 10%. Diagonal elements show the number of SNPs inferred to be associated with each phenotype when the global FDR is controlled at the same level.

	ADHD	ASD	BPD	MDD	SCZ	RA	CD	UC	HDL	T2D	CAD	SBP
ADHD	0	0	0	0	0	0	0	0	0	0	0	0
ASD	0	7	0	0	0	0	0	0	0	0	0	0
BPD	0	0	30	0	0	0	0	0	0	0	0	0
MDD	0	0	0	12	0	0	0	0	0	0	0	0
SCZ	0	0	0	0	270	9	2	1	0	0	2	2
RA	0	0	0	0	9	594	63	71	0	0	0	1
CD	0	0	0	0	2	63	1564	228	30	0	0	1
UC	0	0	0	0	1	71	228	1054	25	0	0	0
HDL	0	0	0	0	0	0	30	25	736	9	3	0
T2D	0	0	0	0	0	0	0	0	9	161	1	0
CAD	0	0	0	0	2	0	0	0	3	1	139	3
SBP	0	0	0	0	2	1	1	0	0	0	3	101

We next considered novel SNPs identified by graph-GPA by evaluating and comparing functional importance between the SNPs identified in separate analyses and by graph-GPA. Specifically, we used the GenoCanyon score [39] that measures functional importance of each genomic locus by integrating functional annotations for conservation, open chromatin, histone modification, and transcription factor binding. For this purpose, we first implemented a lift-over of genomic coordinates to HG19 and annotated with the GenoCanyon annotation file downloaded from its website (http://genocanyon.med.yale.edu/). Fig 2c indicates that the GenoCanyon scores for graph-GPA are larger than 0.5 on average for most phenotypes considered above. Moreover, the GenoCanyon scores for the SNPs identified by graph-GPA are comparable to or higher than those for the SNPs identified in separate analyses. The GenoCanyon scores for GPA are significantly lower than those for graph-GPA, and sometimes even lower than those for separate analysis. We found that the phenotypes with low GenoCanyon scores in GPA analysis correspond to those with weak signals (Tables 1 and 2). This might imply that when signals are weak for certain phenotype, some “bad” combinations of phenotypes might result in false positives for GPA (Table O in S1 Text) because information sharing is allowed between a much smaller number of phenotypes. However, it is often not a trivial task to determine optimal pairs of phenotypes to integrate *a priori*. In contrast, graph-GPA integrates a much larger number of GWAS datasets and this might result in more robust improvement in statistical power in the sense of both sensitivity and specificity, regardless of its signal strength. These results indicate that graph-GPA improves statistical power to identify associated SNPs in a more robust way and the novel genetic variants identified by graph-GPA might also be potentially important ones associated with disease risks.

### graph-GPA identifies bipolar disorder risk associated genetic variants with pleiotropic effects

**Fig 3:**
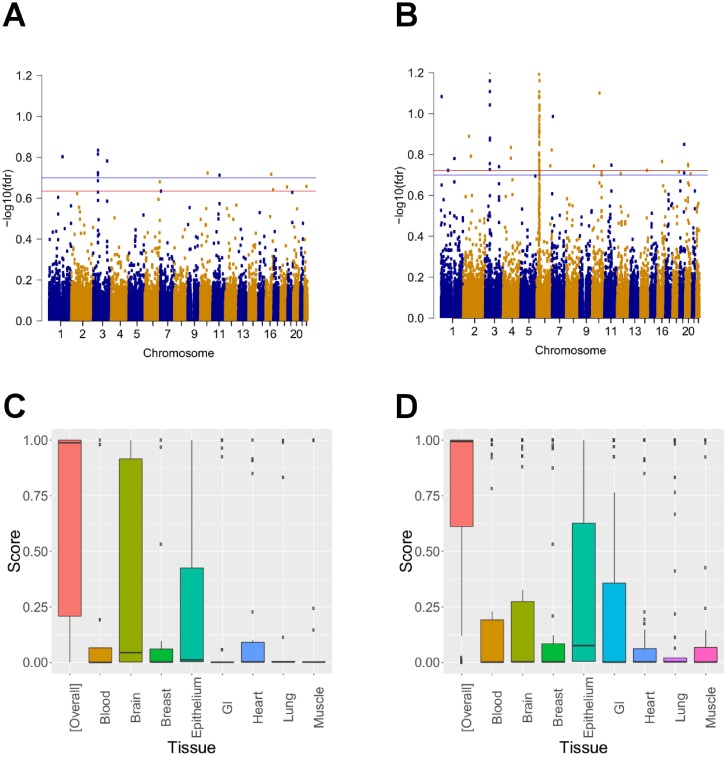
Manhattan plots for association with bipolar disorder (BPD) in separate analysis (a) and joint analysis using graph-GPA (b). Blue and red horizontal lines mean nominal levels for local FDR at 20% and global FDR at 10%, respectively. GeneCanyon ([overall]) and GeneSkyline scores for various tissues for the SNPs associated with BPD, identified in separate analysis (c) and by graph-GPA (d).

Fig 3a and 3b show Manhattan plots for separate analysis and joint analysis using graph-GPA for bipolar disorders (BPD). In the separate analysis under the global FDR level of 10%, we already identified some interesting SNPs, including those located in GNL3, SYNE1, and ANK3 genes, among others. These SNPs were previously identified to be associated with BPD in the literature [10, 40]. For example, GNL3 encodes nucleostemin, which is thought to be a critical regulator of the cell cycle. Aberrant regulation of nucleostemin would be consistent with the neurotrophic hypothesis of mood disorders, which posits that stem-cell proliferative potential in the brain modulates BPD risk [40]. At the same global FDR level, graph-GPA could identify a significantly larger number of novel risk SNPs, especially in chromosomes 3 and 6. Specifically, graph-GPA did not only identify more SNPs located in these genes such as GNL3, but also identified additional biologically interesting SNPs, such as SNPs located in ITIH1 gene in chromosome 3 and multiple SNPs located in the MHC region. These SNPs were also previously identified to be associated with BPD in the literature [10, 40]. For example, ITIH1 encodes a serine protease inhibitor, which is thought to play an anti-inflammatory role [40]. In-depth cross-phenotype investigation indicates that some of these SNPs associated with BPD risk are also related to risk of other diseases, as already suggested by the phenotype graph (Fig 2a). For example, a joint analysis using graph-GPA determined that the GNL3 and ITIH1 genes were also associated with SCZ risk.

**Fig 4:**
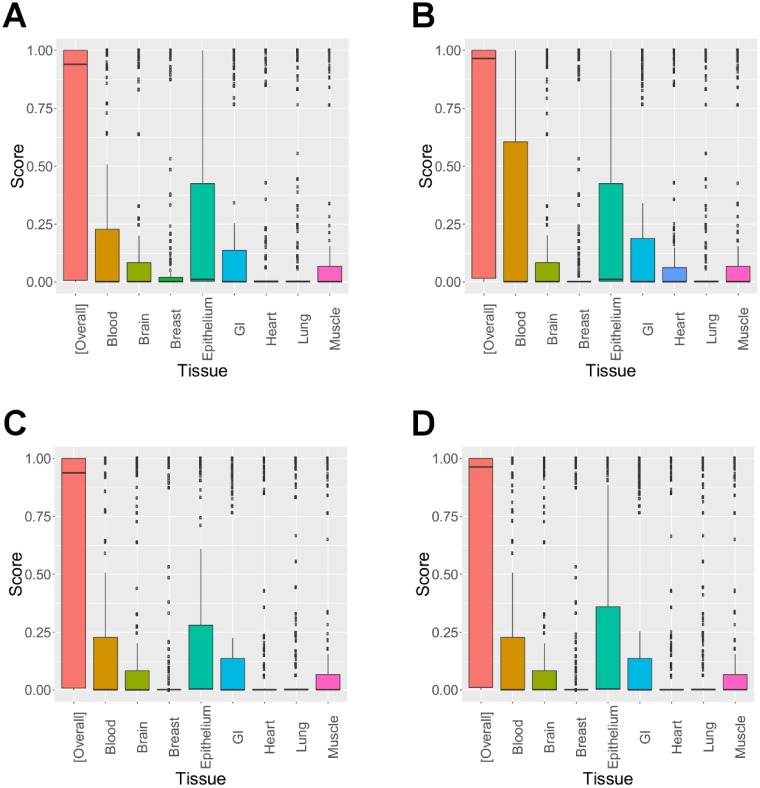
GeneCanyon ([overall]) and GeneSkyline scores for various tissues for the SNPs associated with ulcerative colitis (UC) in separate analysis (a) and in graph-GPA analysis (b), and those associated with Crohn’s disease (CD) in separate analysis (c) and in graph-GPA analysis (d).

We next evaluated overall and tissue-specific functional importance of novel SNPs identified by graph-GPA. Here, in addition to the GenoCanyon scores, we also utilized GenoSkyline scores [41] that measure tissue-specific functional importance of each genomic region, using epigenetic data (H3K4me1, H3K4me3, H3K36me3, H3K27me3, H3K9me3, H3K27ac, H3K9ac, and DNase I hypersensitivity) selected from the Epigenomics Roadmap Project’s 111 consolidated reference epigenomes database. Again, we first implemented a lift-over of genomic coordinates to HG19 and annotated with the GenoSkyline annotation files downloaded from its website (http://genocanyon.med.yale.edu/GenoSkyline/). Fig 3c and 3d indicate that overall functional importance of associated SNPs increased in graph-GPA compared to separate analyses (GenoCanyon). Moreover, the associated SNPs identified by graph-GPA were enriched for more diverse group of tissues. Specifically, while the SNPs identified in a separate analysis were mainly enriched for brain and epithelium, we observed enrichment for blood, brain, epithelium, and gastrointestinal tissues in the associated SNPs identified in graph-GPA analysis (GenoSkyline). Note that although the GenoSkyline score for brain tissue might look lower in Fig 3d compared to Fig 3c, it is actually not the case because graph-GPA identified a significant number of additional SNPs with both high and low scores in brain but simply more SNPs with lower scores in brain were identified by graph-GPA (Figure U in S1 Text). We observed similar enrichment patterns for other psychiatric disorders as well (ASD, MDD, and SCZ; Figures M–O in S1 Text). We note that the SNPs associated with autoimmune diseases are specifically enriched for blood, epithelium, and gastrointestinal tissues (Fig 4 and Figure P in S1 Text), as previously reported [41]. This might imply that graph-GPA identified more pleiotropic genetic variants that are potentially associated with both bipolar disorder and autoimmune diseases. This is again consistent with our observation of genetic correlation between psychiatric disorders and autoimmune diseases (Fig 2a).

### graph-GPA suggests significant genetic sharing among autoimmune diseases

We first investigated the genes that were previously reported to be associated with both CD and UC, which are collectively called as inflammatory bowel disease (IBD) [42]. Separate analysis at the global FDR level of 10% could identify some of these genes, including KIF21B, BRE, IL18RAP, DAP, NKX2-3, IL23R, SMAD3, JAK2, CREM, STAT3, TYK2, REL, CCR6, and TYK2. Joint analysis using graph-GPA at the same FDR level identified comparable number of additional IBD-risk associated genes, including IL10, FOSL2, FCGR2A, STAT4, NDFIP1, ORMDL3, IL2RA, MAP3K8, and CD226. These results indicate that the proposed graph-GPA model can potentially improve statistical power to identify risk associated genetic variants by leveraging pleiotropy structure. In order to further validate novel genetic variants identified by graph-GPA, we compared KEGG pathways enriched for the SNPs identified in separate analyses and those identified only by graph-GPA, using the DAVID functional annotation tool (https://david.ncifcrf.gov/). As expected, for each of CD, UC, and RA, multiple pathways related to autoimmune diseases (antigen processing and presentation, allograft rejection, type I diabetes mellitus, and autoimmune thyroid disease, among others) are enriched for both the SNPs identified in separate analyses and those identified by graph-GPA. In addition, these pathways are also enriched for the SNPs associated with both RA and CD, and for the SNP associated with both RA and UC (regardless of separate or joint analyses). Moreover, cardiomyopathy pathways are enriched for both the SNPs identified in separate analyses and the SNPs identified only by graph-GPA for IBD (CD and UC). Various signaling pathways (GnRH signaling pathway and calcium signaling pathway, among others) are also enriched for the SNPs identified in separate analyses for CD and the SNPs identified only by graph-GPA for UC. Finally, we evaluated overall and tissue-specific functional importance of novel SNPs using the GenoCanyon and GenoSkyline scores for these SNPs. Fig 4 and Figure P in S1 Text show the results for UC, CD, and RA, and they indicate that overall functional importance of the SNPs identified by graph-GPA is comparable to the SNPs identified in separate analyses (GenoCanyon). Moreover, the SNPs associated with each of UC, CD, and RA are enriched for blood, epithelium, and gastrointestinal tissues in both separate analyses and joint analyses using graph-GPA (GenoSkyline), which is consistent with the previous findings [41]. These results indicate that the novel SNPs identified by graph-GPA share similar functions with those identified in separate analyses for autoimmune diseases, and this implies that these novel genetic variants identified by graph-GPA are potentially associated with risk of these autoimmune diseases.

## Discussion

Here we first briefly discuss some key assumptions made in the graph-GPA framework. First, in the current paper, we assumed the standard normal distribution to model probit transformed *p*-values for background SNPs, which is equivalent to the uniformity assumption of *p*-values for background SNPs (i.e., theoretical null distribution assumption). While this assumption is mathematically justified [18] and also works well in practice as shown in its application to GWAS data for 12 phenotypes, it is still important to confirm that the *p*-values used as an input for graph-GPA reasonably satisfy this assumption. For example, the distribution assumption for background SNPs can be violated and type I errors of graph-GPA can be inflated if population stratification and cryptic relatedness are not properly taken into account. Hence, these confounding effects should be checked carefully and addressed before applying graph-GPA to the *p*-values. Second, in the proposed model, genetic variants were assumed to be independent. This independence assumption greatly simplifies our model structure and results in efficient computation, which allows practical use of the graph-GPA framework. However, in real GWAS data, genetic variants are often correlated due to linkage disequilibrium (LD) [43]. In practice, this issue can be addressed by promoting independence among genetic variants by LD pruning approaches, for example. Third, in the proposed model, it is assumed that the edge in the phenotype graph is purely due to genetic correlation between these phenotypes. However, when some subjects are shared between genetic studies, this can potentially generate artificial correlation among phenotypes. We investigated the impact of overlapping subjects on the estimation of phenotype graph using simulation data (Section 17 of S1 Text). The results indicate that the phenotype graph estimation of graph-GPA is robust to substantial sharing of subjects among genetic studies. However, we still recommend to check this issue carefully when using the proposed graph-GPA approach.

In spite of great successes of the proposed graph-GPA framework, it can be further improved as follows. First, in this paper, we assumed a uniform distribution over all possible subgraphs as a prior distribution for the phenotype graph. While this approach already provided a parsimonious graph that is well supported by the literature, it can be further improved by utilizing other related information useful to construct a phenotype graph. Second, in our real data analysis with the GWAS data for 12 phenotypes, we conservatively generated 50,000 MCMC iterations to guarantee the chain’s mixing and convergence. In this setting, our MCMC algorithm ran in a reasonable time, i.e., about three computation days on a standard single-core desktop machine. However, in practice, a significantly smaller number of iterations can be used after checking the chain’s convergence. For example, we re-analyzed our real data by generating 10,000 MCMC iterations, which took about 16 computation hours, and obtained inference results that were very similar to those generated from the five-time longer run of the MCMC chain. However, we note that as the number of phenotypes increases, the computation time will also increase dramatically. In order to improve the scalability of the proposed method, we plan to implement parallel computing algorithms for cluster and GPU computing. Specifically, in our MCMC steps for posterior inference (Section 1 of S1 Text), the update for **e_t_** (Step S1), which is the most computationally expensive part, can be easily updated in parallel across SNPs because the conditional distribution of **e_t_** given (*α*, *β*, **G**) is independent of that of $e_{t\prime }$ when *t* ≠ *t*′ in our model. In addition, more computationally efficient algorithms beyond standard MCMC can also be considered, including advanced Monte Carlo techniques such as Hamiltonian MCMC [44] and simulated annealing [45, 46], and approximated calculation approaches such as pseudo likelihood [47] and variational Bayes [48].

In summary, we proposed graph-GPA, a novel statistical framework to integrate GWAS datasets for multiple phenotypes using a hidden MRF approach. By effectively sharing information across multiple GWAS datasets for genetically related phenotypes, the proposed statistical model improves statistical power to identify risk-associated genetic variants. In addition, the proposed method provides a parsimonious graph representing genetic relationship among a large number of phenotypes. Our simulation studies indicate that graph-GPA has potential to prioritize genetically more related pairs of phenotypes and to improve statistical power to identify risk-associated genetic variants, compared to both separate analysis and the statistical method integrating smaller number of GWAS datasets. In our application of graph-GPA to GWAS datasets for twelve phenotypes, we observed that clinically related phenotypes are tightly linked in the estimated phenotype graph, while edges across different groups of phenotypes could also be supported by multiple literature. At the same nominal false discovery rate, joint analyses of graph-GPA could identify significantly larger number of additional genetic variants associated with each phenotype and those with pleiotropic effects. The pathway analysis and functional analysis of these novel genetic variants indicate that these novel variants might potentially have functional importance. We expect that graph-GPA would provide a powerful approach for prioritizing risk-associated genetic variants and elucidating the pleiotropic architecture of complex traits, which can contribute to a better understanding of shared genetic mechanisms and the development of improved diagnosis and therapeutics.

## Supporting information

S1 Text Supporting information for graph-GPA. Details about posterior inference and additional results.
(PDF)
