# Supplementary material for: graph-GPA: A graphical model for prioritizing GWAS results and investigating pleiotropic architecture
Source: PLoS Comput Biol. 2017 Feb 17;13(2):e1005388. doi: 10.1371/journal.pcbi.1005388 (PMC5347371; doi:10.1371/journal.pcbi.1005388)
Supplement: S1 Text — Details about posterior inference and additional results. (PDF) [file pcbi.1005388.s001.pdf]

Text S1: Supporting information for "graph-GPA: A Graphical Model for Prioritizing GWAS Results and Investigating Pleiotropic Architecture"

Dongjun Chung[1,@], Hang J. Kim[2], Hongyu Zhao[3,4,5,6]

1 Department of Public Health Sciences, Medical University of South Carolina, Charleston, SC, USA.
2 Department of Mathematical Sciences, University of Cincinnati, Cincinnati, OH, USA.
3 Department of Biostatistics, Yale School of Public Health, New Haven, CT, USA.
4 Program in Computational Biology and Bioinformatics, Yale University, New Haven, CT, USA.
5 Department of Genetics, Yale School of Medicine, West Haven, CT, USA.
6 VA Cooperative Studies Program Coordinating Center, West Haven, CT, USA.

@ Correspondence should be addressed to Dongjun Chung (chungd@musc.edu).

Contents

1 MCMC steps for posterior inferences **3**

2 Simulation studies: Parameter estimation performance **5**

3 Simulation studies: False discovery rate control performance **6**

4 Simulation studies: Association mapping **7**

5 Simulation studies: Receiver operating characteristic curves **9**

6 GWAS of 12 phenotypes: Evaluation of the standard normal assumption for background SNPs **11**

7 GWAS of 12 phenotypes: Evaluation of the log-normal assumption for associated SNPs **15**

8 GWAS of 12 phenotypes: Alternative emission distribution for associated SNPs **19**

9 GWAS of 12 phenotypes: Convergence diagnostics **21**

10 GWAS of 12 phenotypes: Model robustness to prior distributions **22**

11 GWAS of 12 phenotypes: Graph estimation **24**

12 GWAS of 12 phenotypes: graph-GPA analysis using RA sub-cohorts **25**

13 GWAS of 12 phenotypes: graph-GPA analysis with less stringent FDR controls **28**

14 GWAS of 12 phenotypes: GPA analysis **29**

15 GWAS of 12 phenotypes: GenoCanyon and GenoSkyline annotation of the graph-GPA analysis results **30**

16 GWAS of Bipolar Disorder: GenoSkyline annotation of the graph-GPA analysis results for brain tissue **35**

17 Impact of overlapping subjects on the estimation of pleiotropic architecture **36**

1 MCMC steps for posterior inferences

The posterior inferences are implemented by the following MCMC steps:

S1. For each i and t, draw $e_{it} \sim \text{Bernoulli}(p_1^*)$ where

$$p_1^* = \frac{\exp\left(\alpha_i + \sum_{j\sim i} \beta_{ij} e_{jt}\right) \cdot p(y_{it}|e_{it}=1, \mu_i, \sigma_i^2)}{\sum_{e^*\in\{0,1\}} \exp\left(\alpha_i e^* + \sum_{j\sim i} \beta_{ij}\ e^*\ e_{jt}\right) \cdot p(y_{it}|e_{it}=e^*, \mu_i, \sigma_i^2)}.$$

S2. For each i, draw μ_i from its full conditional distribution,

$$\mu_i \sim \text{N}\left(\frac{\sigma_i^2\theta_\mu + \tau_\mu^2 \sum_{\{t:e_{it}=1\}} \log y_{it}}{\sigma_i^2 + \tau_\mu^2 n_i}, \frac{\sigma_i^2 \tau_\mu^2}{\sigma_i^2 + \tau_\mu^2 n_i}\right)$$

where $n_i = \#\{t : e_{it} = 1\}$.

S3. For each i, draw σ_i^2 from its full conditional distribution,

$$\sigma_i^2 \sim \text{IG}\left(a_\sigma + \frac{n_i}{2}, b_\sigma + \frac{\sum_{\{t:e_{it}=1\}} (\log y_{it} - \mu_i)^2}{2}\right)$$

where $n_i = \#\{t : e_{it} = 1\}$

S4. For each i, update α_i with the Metropolis-Hastings:

1. Propose α_i^q from $q(\alpha_i^q|\alpha_i) = \text{N}(\alpha_i^q; \alpha_i, s_\alpha^2)$. We set $s_\alpha = 0.1$.
2. Update $\alpha_i = \alpha_i^q$ with the acceptance probability

$$\min\left[1, \left\{\prod_{t=1}^{T} \frac{\text{C}(\boldsymbol{\alpha}, \boldsymbol{\beta}, \boldsymbol{G})}{\text{C}(\boldsymbol{\alpha}^q, \boldsymbol{\beta}, \boldsymbol{G})} \frac{\exp(\alpha_i^q e_{it})}{\exp(\alpha_i e_{it})}\right\} \frac{p(\alpha_i^q)}{p(\alpha_i)}\right]$$

where $\boldsymbol{\alpha}^q = (\alpha_1, \ldots, \alpha_{i-1}, \alpha_i^q, \alpha_{i+1}, \ldots, \alpha_n)$.

S5. For each (i, j) such that $E(i, j) = 1$, update β_{ij} with the Metropolis-Hastings:

1. Propose β_{ij}^q from the truncated normal proposal distribution bounded above zero, $q(\beta_{ij}^q|\beta_{ij}) = \text{N}_+(\beta_{ij}^q; \beta_{ij}, s_\beta^2)$. We set $s_\beta = 0.1$.
2. Update $\beta_{ij} = \beta_{ij}^q$ with the acceptance probability

$$\min\left[1, \left\{\prod_{t=1}^{T} \frac{\text{C}(\boldsymbol{\alpha}, \boldsymbol{\beta}, \boldsymbol{G})}{\text{C}(\boldsymbol{\alpha}, \boldsymbol{\beta}^q, \boldsymbol{G})} \frac{\exp(\beta_{ij}^q e_{it} e_{jt})}{\exp(\beta_{ij} e_{it} e_{jt})}\right\} \frac{p(\beta_{ij}^q|E(i,j))}{p(\beta_{ij}|E(i,j))}\ \frac{q(\beta_{ij}|\beta_{ij}^q)}{q(\beta_{ij}^q|\beta_{ij})}\right]$$

where $\boldsymbol{\beta}^q = (\beta_{12}, \beta_{13}, \ldots, \beta_{i,j-1}, \beta_{ij}^q, \beta_{i,j+1}, \ldots, \beta_{n-1,n-2}, \beta_{n-1,n})$.

S6. For a randomly chosen (i, j), update $(\beta_{ij}, \boldsymbol{G})$ by the reversible jump process:

1. Let $|E|$ denote the number of edges in the current graph $\boldsymbol{G}$, i.e., $|E| = \sum_{\{(i,j):i\neq j\}} E(i, j)$. Propose the number of edges E^q from the proposal distribution,

$$q(E^q \mid |E|) = 0.5\ I\left[E^q = |E| - 1\right] + 0.5\ I\left[E^q = |E| + 1\right].$$

   If $|E| = 0$, $E^q = 1$ with probability 1. If $|E| = |E|_{\max}$, $E^q = |E|_{\max} - 1$ with probability 1 where $|E|_{\max}$ denotes the maximum number of possible edges, i.e., $|E|_{\max} = \binom{n}{2}$.

2. Propose $\boldsymbol{G}^q$ from the proposal distribution $q(\boldsymbol{G}^q|\boldsymbol{G}, E^q)$ and then β^q_{ij} from the proposal distribution $q(\beta^q_{ij}|\boldsymbol{G}^q, E^q)$.

   (a) For the case where $E^q > |E|$, randomly select a pair of (i, j) such that $E(i, j) = 0$ and let $E(i, j)^q = 1$ with the proposal distribution

$$q(\boldsymbol{G}^q|\boldsymbol{G}, E^q) = \frac{1}{\#\{(i^*, j^*) : G_{i^*j^*} = 0\}} = \frac{1}{|E|_{\max} - |E|}$$

   while $G^q_{i^*j^*} = G_{i^*j^*}$ for all other (i^*, j^*). Propose β^q_{ij} from $q(\beta^q_{ij}|E(i, j)^q, E^q) = \Gamma(\beta^q_{ij}; a_{\beta_G}, b_{\beta_G})$. We set $a_{\beta_G} = b_{\beta_G} = 1$.

   (b) For the case where $E^q < |E|$, randomly select a pair of (i, j) such that $E(i, j) = 1$ and let $E(i, j)^q = 0$ with the proposal distribution

$$q(\boldsymbol{G}^q|\boldsymbol{G}, E^q) = \frac{1}{\#\{(i^*, j^*) : G_{i^*j^*} = 1\}} = \frac{1}{|E|}$$

   while $G^q_{i^*j^*} = G_{i^*j^*}$ for all other (i^*, j^*). Propose β^q_{ij} from $q(\beta^q_{ij}|E(i, j)^q, E^q) = \delta_0(\beta^q_{ij})$.

3. Update $(\beta_{ij}, \boldsymbol{G}) = (\beta^q_{ij}, \boldsymbol{G}^q)$ with the acceptance probability

$$\min\left[1, \left\{\prod_{t=1}^{T} \frac{\mathrm{C}(\boldsymbol{\alpha}, \boldsymbol{\beta}, \boldsymbol{G})}{\mathrm{C}(\boldsymbol{\alpha}, \boldsymbol{\beta}^q, \boldsymbol{G}^q)} \frac{\exp(\beta^q_{ij} e_{it} e_{jt})}{\exp(\beta_{ij} e_{it} e_{jt})}\right\} \frac{p(\beta^q_{ij}|E(i, j)^q) p(\boldsymbol{G}^q)}{p(\beta_{ij}|E(i, j)) p(\boldsymbol{G})} \frac{q(\beta_{ij}|\boldsymbol{G}, |E|) q(\boldsymbol{G}|\boldsymbol{G}^q, |E|) q(|E||E^q)}{q(\beta^q_{ij}|\boldsymbol{G}^q, E^q) q(\boldsymbol{G}^q|\boldsymbol{G}, E^q) q(E^q||E|)}\right]$$

   where $\boldsymbol{\beta}^q = (\beta_{12}, \beta_{13}, \ldots, \beta_{i,j-1}, \beta^q_{ij}, \beta_{i,j+1}, \ldots, \beta_{n-1,n-2}, \beta_{n-1,n})$ and $\boldsymbol{G}^q = (G_{12}, G_{13}, \ldots, G_{i,j-1}, E(i, j)^q, G_{i,j+1}, \ldots, G_{n-1,n-2}, G_{n-1,n})$.

   Note that $p(\beta_{ij}|E(i, j)) = q(\beta_{ij}|\boldsymbol{G}, |E|)$ when $E^q > q$ and $p(\beta^q_{ij}|E(i, j)^q) = q(\beta^q_{ij}|\boldsymbol{G}^q, E^q)$ when $E^q < q$ and, so they are cancelled out from the acceptance probability.

2 Simulation studies: Parameter estimation performance

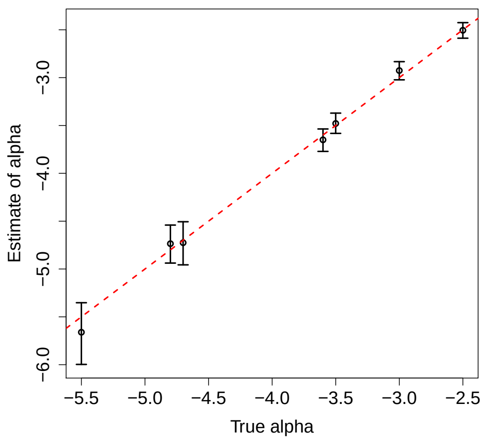

(a) Comparison of true vs. estimated α_i.

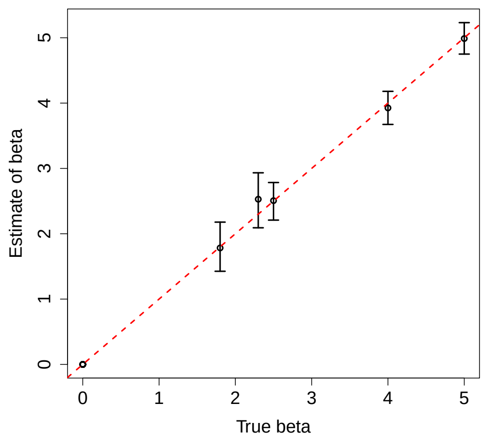

(b) Comparison of true vs. estimated β_{ij}.

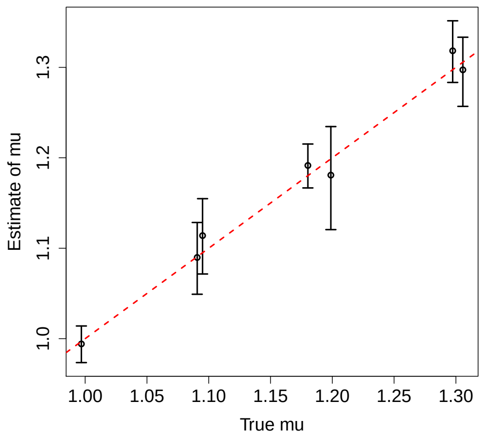

(c) Comparison of true vs. estimated μ_i.

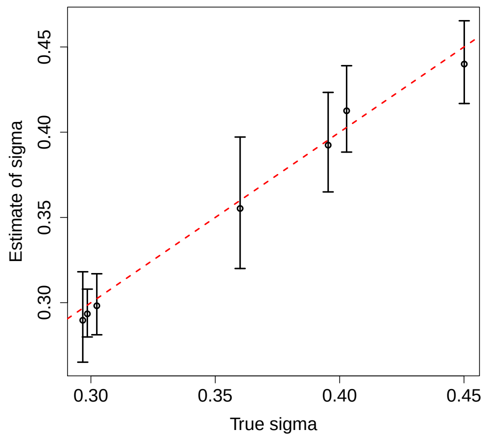

(d) Comparison of true vs. estimated σ_i.

Figure A: Simulation studies: Parameter estimation performance. The dots represent point estimates with their 95% credible intervals represented by the bars. Note that true values (x-axis) were jittered for better visualization in (c) and (d).

3 Simulation studies: False discovery rate control performance

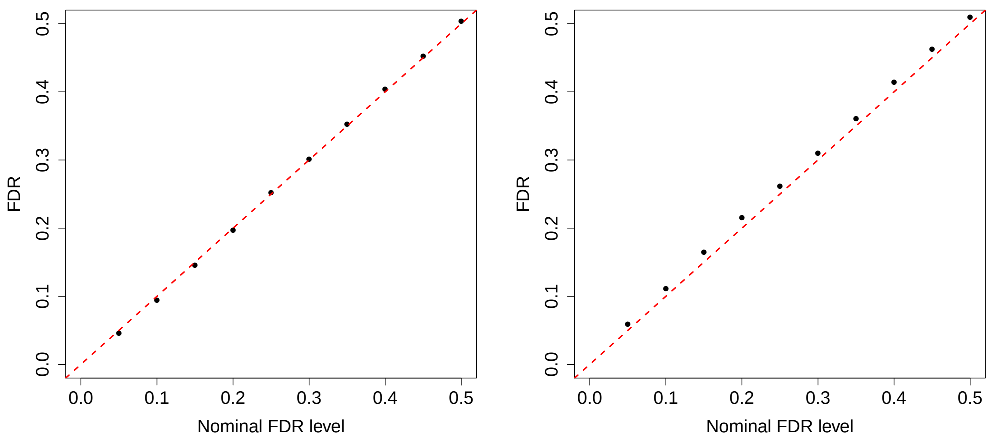

(a) Nominal FDR level vs. FDR for P1. (b) Nominal FDR level vs. FDR for P7.

Figure B: Simulation studies: False discovery rate control performance of graph-GPA. Phenotype P1 (a) represents a phenotype that is highly genetically correlated with other phenotypes while phenotype P7 (b) represents an independent phenotype. False discovery rates were well controlled at various nominal levels in both cases.

4 Simulation studies: Association mapping

Table A: Simulation studies (joint analysis using graph-GPA): Numbers of SNPs identified to be associated with each pair of phenotypes by controlling the global FDR at nominal level of 10%. Diagonal elements show the number of SNPs inferred to be associated with each phenotype when the global FDR is controlled at the same level.

	P1	P2	P3	P4	P5	P6	P7
P1	692	591	170	81	77	29	7
P2	591	1339	219	114	114	52	22
P3	170	219	285	111	93	14	3
P4	81	114	111	913	767	34	19
P5	77	114	93	767	1199	39	20
P6	29	52	14	34	39	1102	19
P7	7	22	3	19	20	19	558

Table B: Simulation studies (joint analysis using GPA): Numbers of SNPs identified to be associated with each pair of phenotypes by controlling the global FDR at nominal level of 10%. Diagonal elements show the number of SNPs inferred to be associated with each phenotype when the global FDR is controlled at the same level. Note that for the diagonal elements, we show the number of associated SNPs averaged over the pairs estimated to be correlated with each phenotype (P1 - P5) or over all possible pairs (P6 and P7).

	P1	P2	P3	P4	P5	P6	P7
P1	628	697	164	38	34	19	3
P2	697	1270	245	36	53	29	14
P3	164	245	222	99	60	8	2
P4	38	36	99	941	1084	27	15
P5	34	53	60	1084	1193	40	12
P6	19	29	8	27	40	1081	16
P7	3	14	2	15	12	16	560

Table C: Simulation studies (separate analysis): Numbers of SNPs identified to be associated with each pair of phenotypes by controlling the global FDR at nominal level of 10%. Diagonal elements show the number of SNPs inferred to be associated with each phenotype when the global FDR is controlled at the same level.

	P1	P2	P3	P4	P5	P6	P7
P1	497	118	43	25	27	19	5
P2	118	1055	34	27	46	31	17
P3	43	34	187	42	26	7	12
P4	25	27	42	761	362	27	16
P5	27	46	26	362	1059	38	15
P6	19	31	7	27	38	1102	19
P7	5	17	2	16	15	19	558

5 Simulation studies: Receiver operating characteristic curves

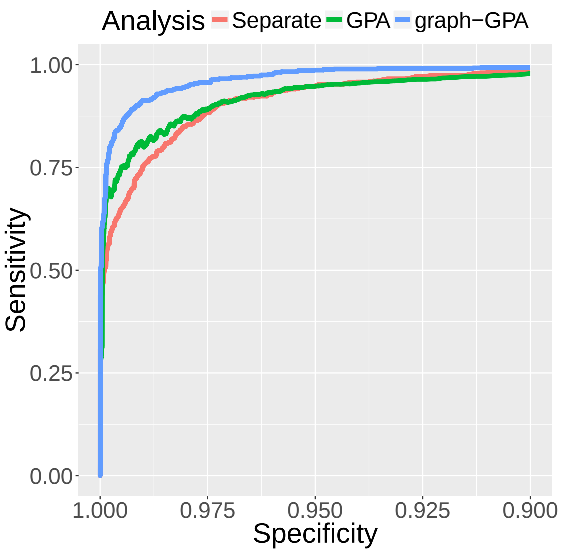

(a) P1.

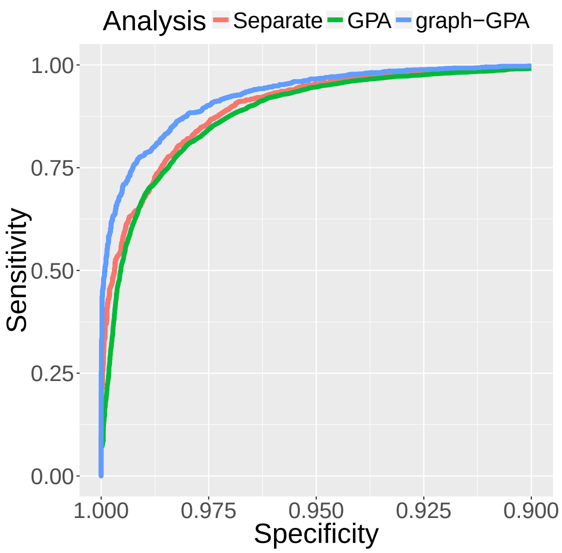

(b) P2.

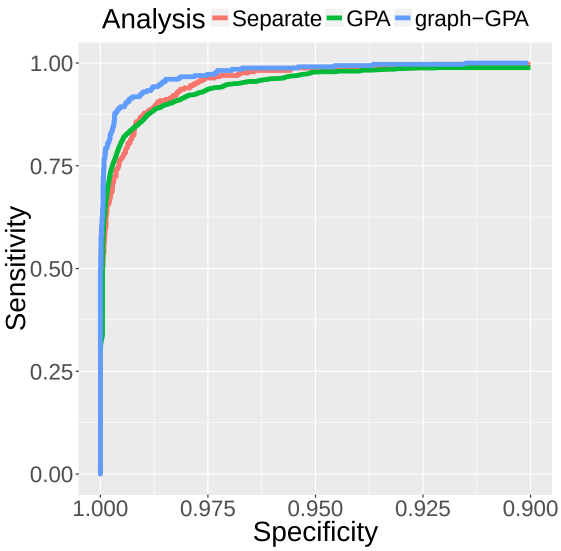

(c) P3.

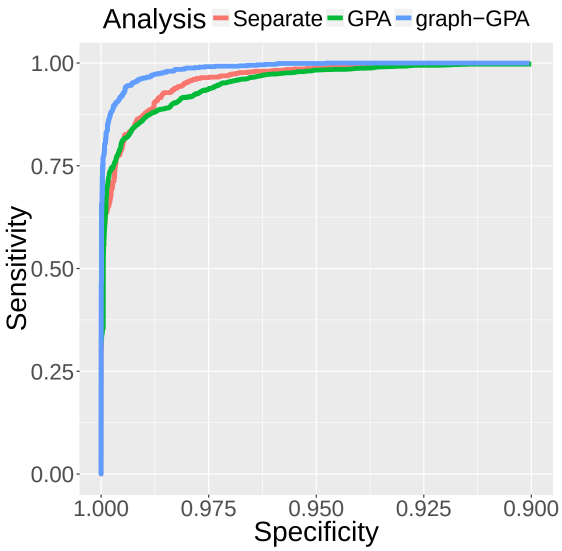

(d) P4.

Figure C: Simulation studies: Receiver operating characteristic (ROC) curves for phenotypes P1 – P4.

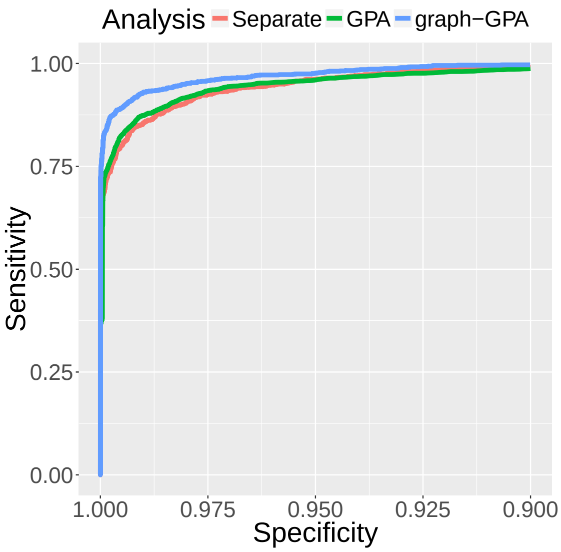

(a) P5.

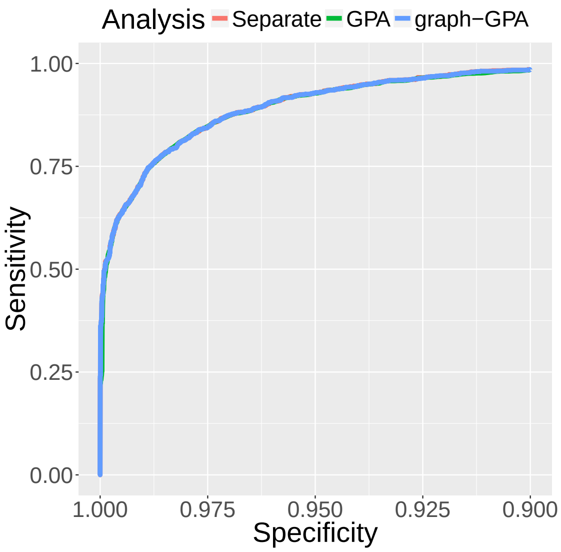

(b) P6.

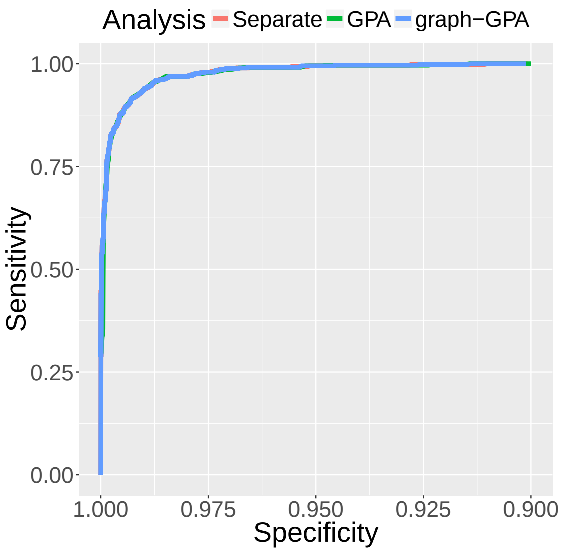

(c) P7.

Figure D: Simulation studies: Receiver operating characteristic (ROC) curves for phenotypes P5 – P7.

6 GWAS of 12 phenotypes: Evaluation of the standard normal assumption for background SNPs

In order to confirm the appropriateness of the theoretical null distribution assumption, we implemented exploratory analyses of real GWAS data. Specifically, we first determined "background SNPs" for each GWAS data using the criterion that the local FDR of a SNP is larger than 0.50. Then, we compared the histogram of transformed p-values for these background SNPs with N(0,1). In addition, we also statistically evaluated the violation of theoretical null distribution assumption using Sharpiro-Wilk test (using the `R` implementation `shapiro.test()`). These results are provided in Figures E $-$ G and they confirmed that there is no significant violation of the theoretical null distribution assumption.

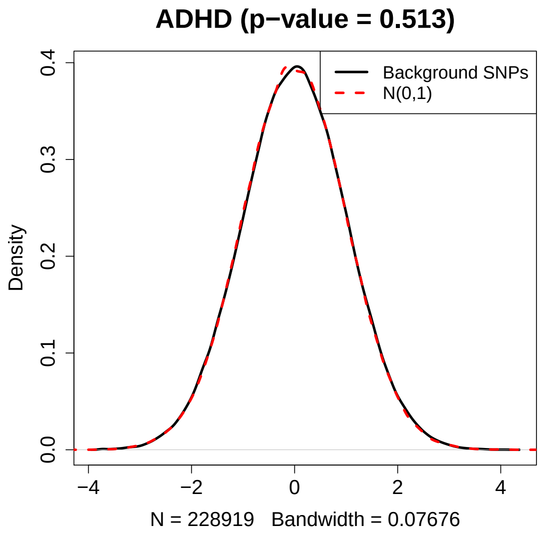

(a) ADHD.

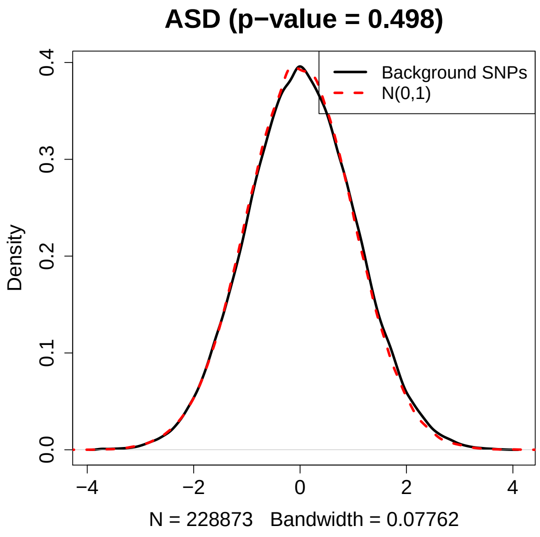

(b) ASD.

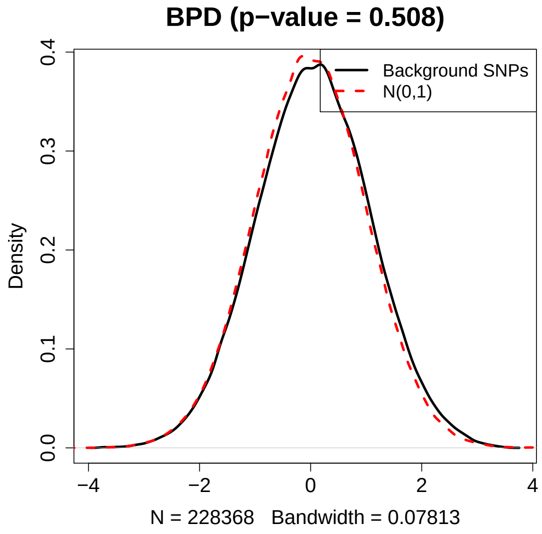

(c) BPD.

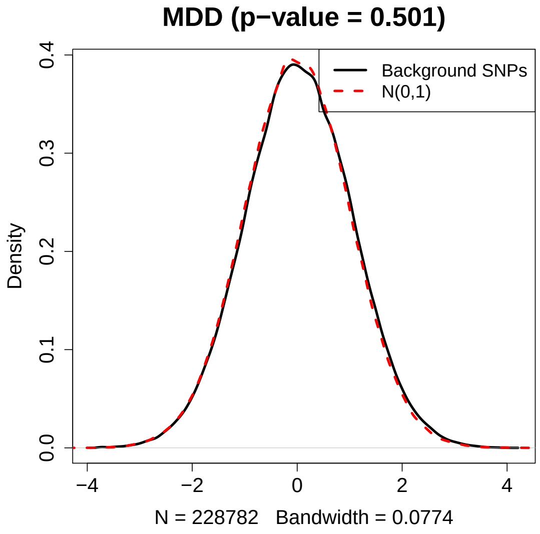

(d) MDD.

Figure E: Histogram of transformed p-values for background SNPs, overlaid with standard normal distribution, for phenotypes ADHD, ASD, BPD, and MDD.

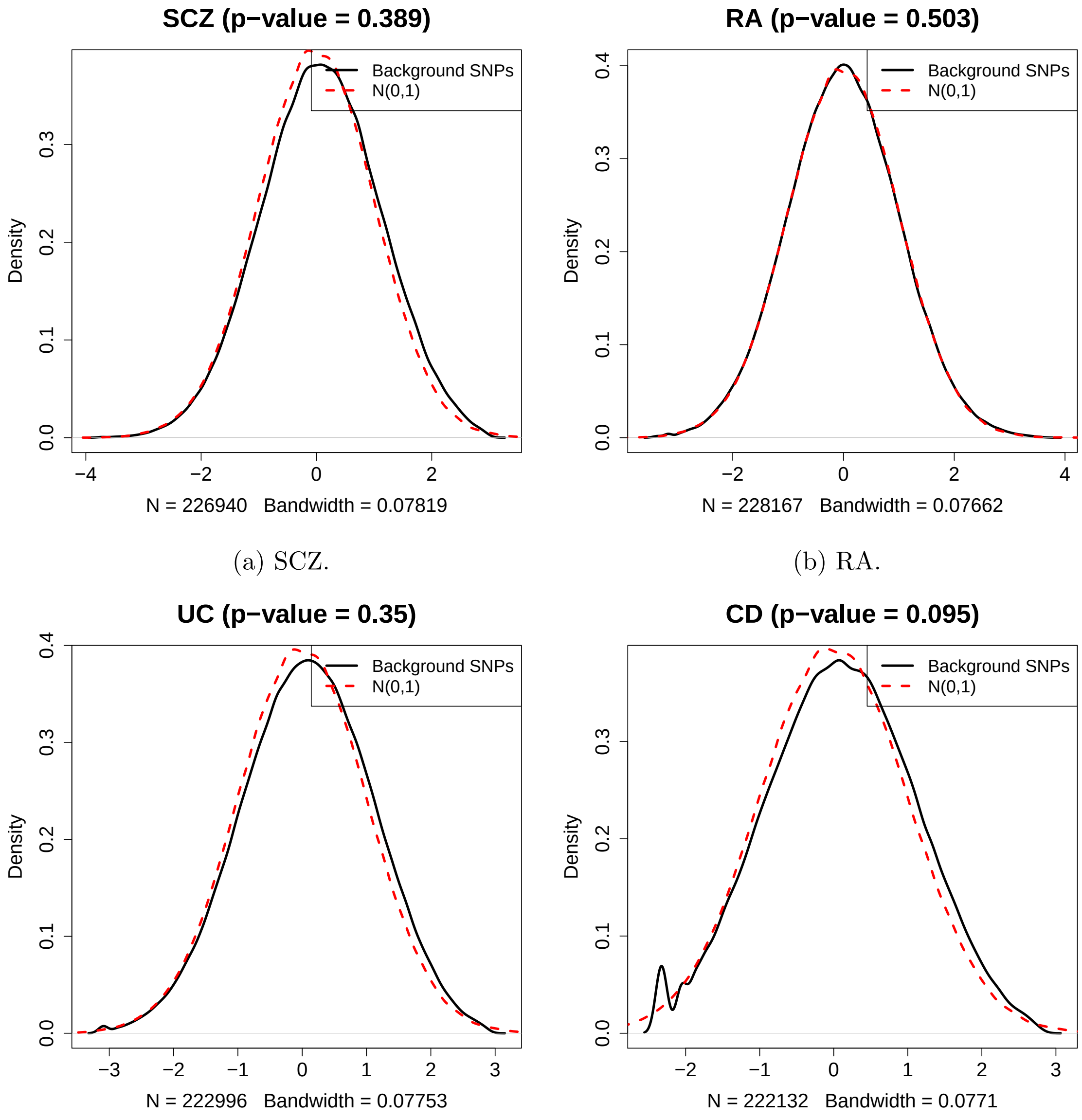

(a) SCZ.

(b) RA.

(c) UC.

(d) CD.

Figure F: Histogram of transformed p-values for background SNPs, overlaid with standard normal distribution, for phenotypes SCZ, RA, UC, and CD.

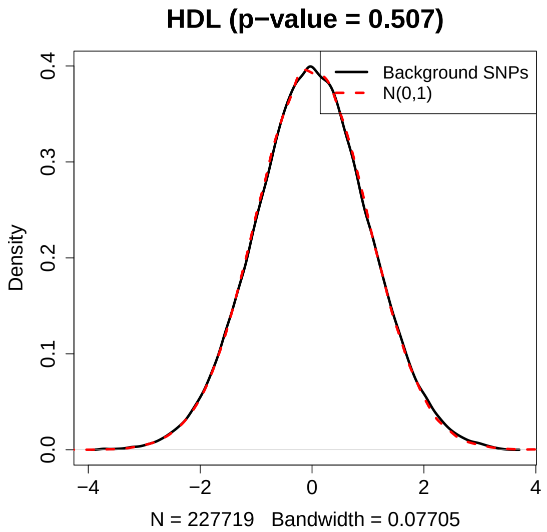

(a) HDL.

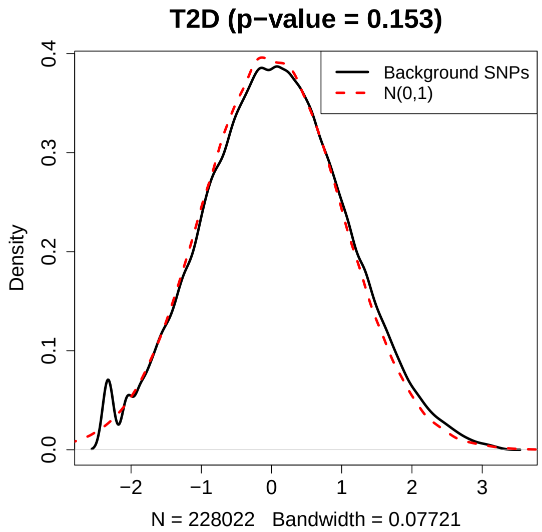

(b) T2D.

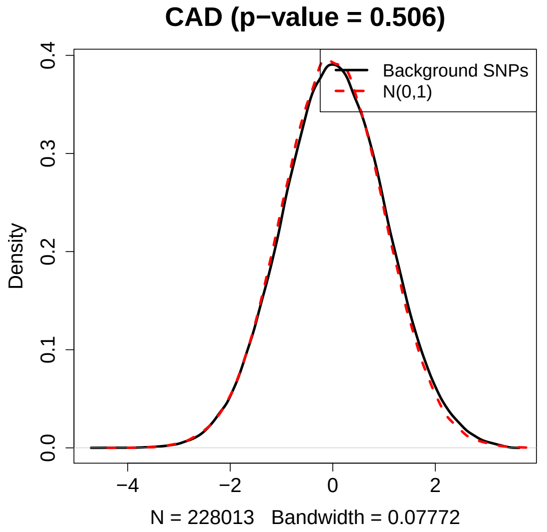

(c) CAD.

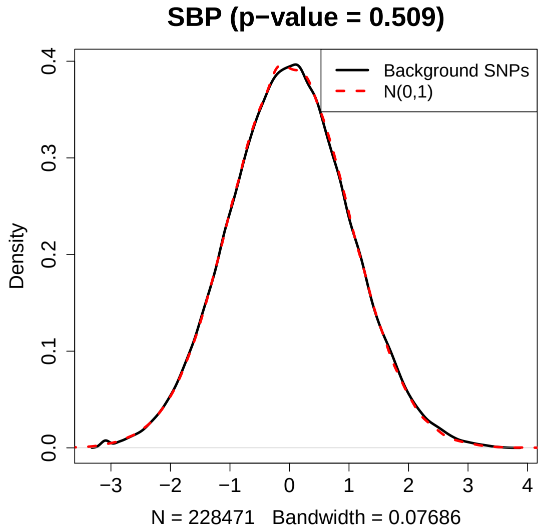

(d) SBP.

Figure G: Histogram of transformed p-values for background SNPs, overlaid with standard normal distribution, for phenotypes HDL, T2D, CAD, and SBP.

7 GWAS of 12 phenotypes: Evaluation of the log-normal assumption for associated SNPs

In order to confirm the appropriateness of the log-normal distribution as the non-null distribution, we implemented a posterior predictive checking, i.e., compared the distribution of tranformed p-values with those simulated from the fitted graph-GPA model. These posterior predictive checking results for real data are provided in Figures H $-$ J. They indicate that the proposed model (i.e., a mixture of standard normal and log-normal distributions) fits the data nicely, which confirms the appropriateness of using log-normal distribution as the non-null distribution.

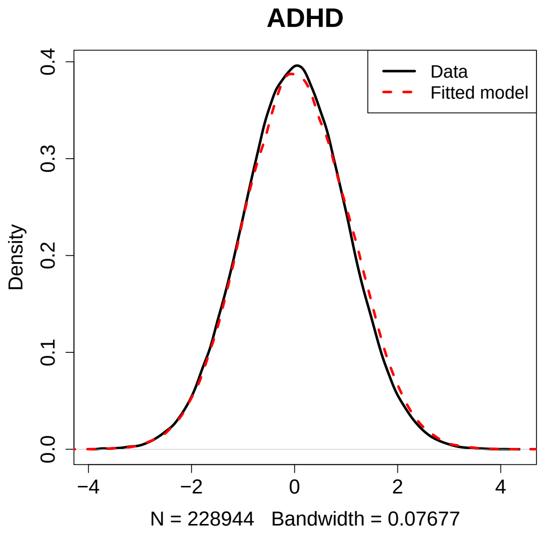

(a) ADHD.

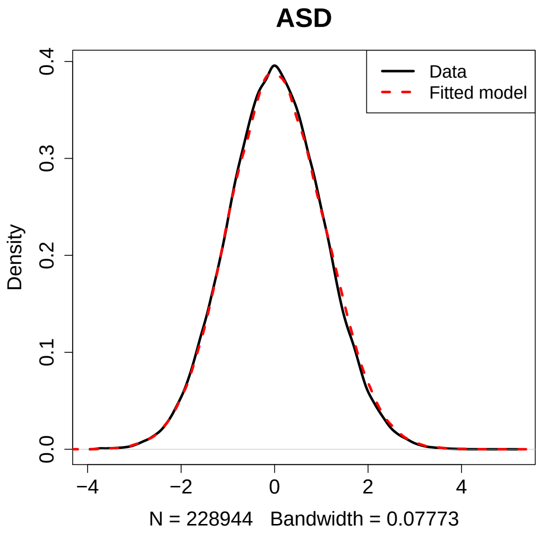

(b) ASD.

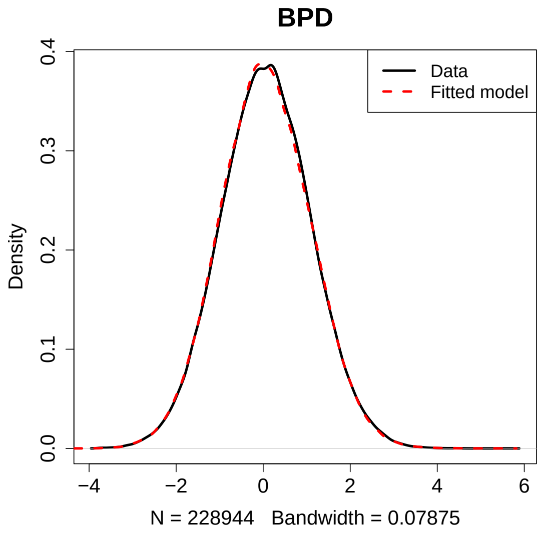

(c) BPD.

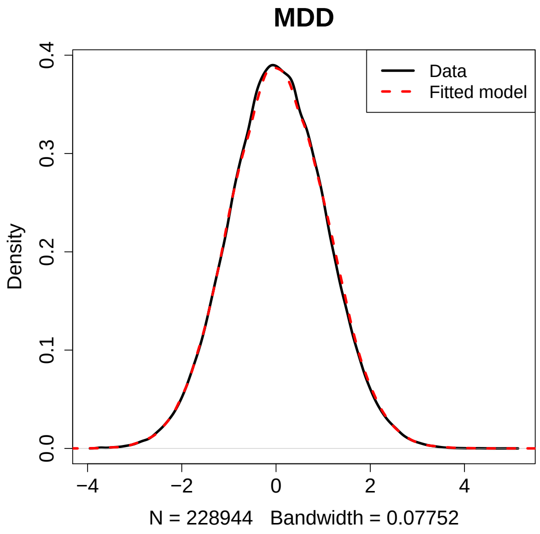

(d) MDD.

Figure H: Posterior predictive checking results for phenotypes ADHD, ASD, BPD, and MDD.

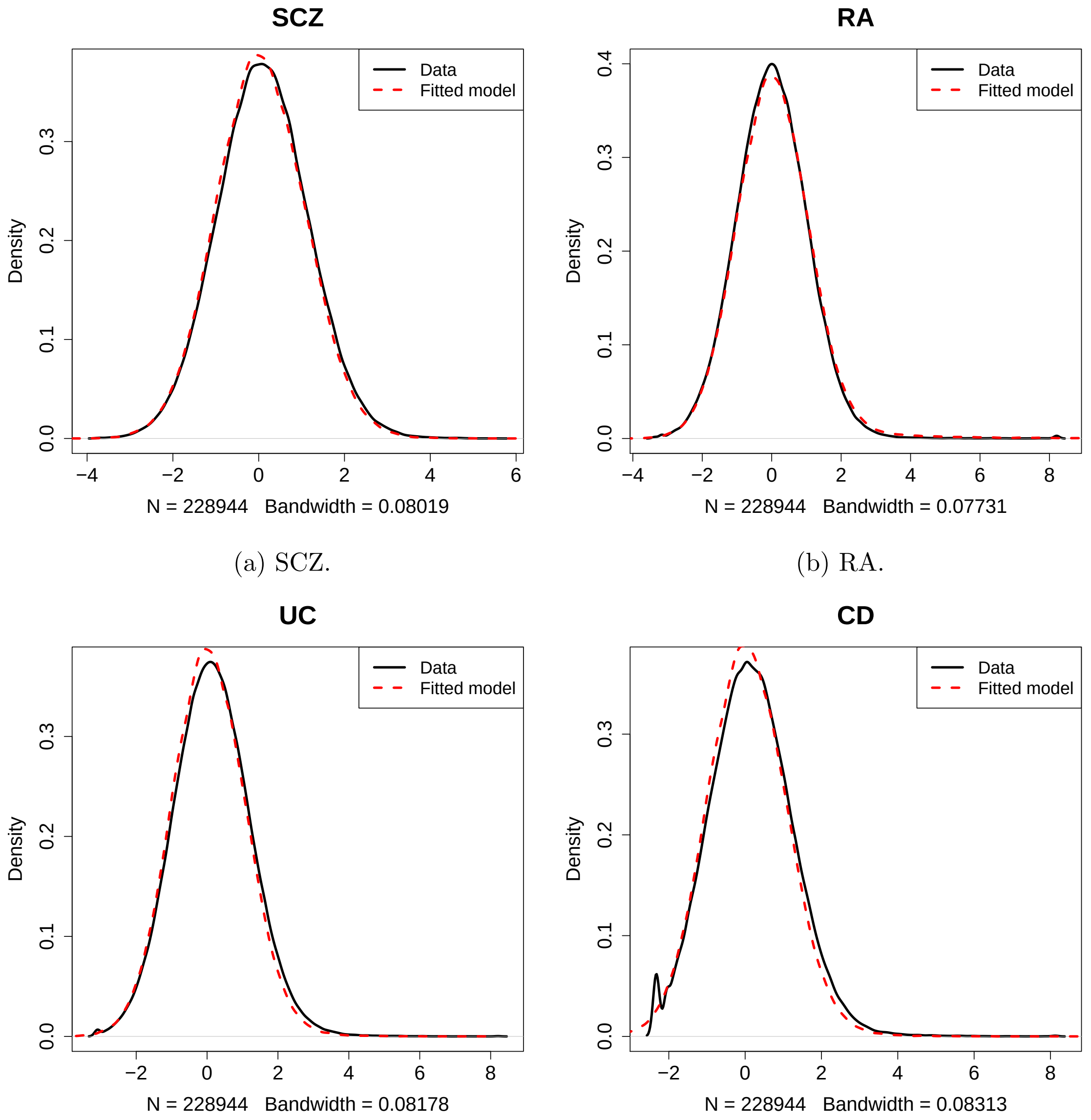

(a) SCZ.

(b) RA.

(c) UC.

(d) CD.

Figure I: Posterior predictive checking results for phenotypes SCZ, RA, UC, and CD.

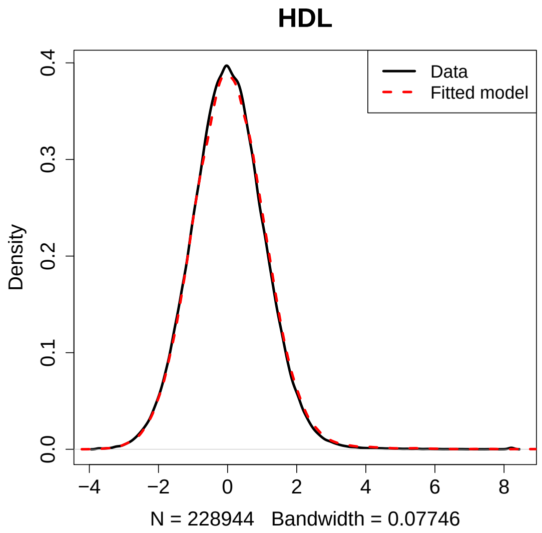

(a) HDL.

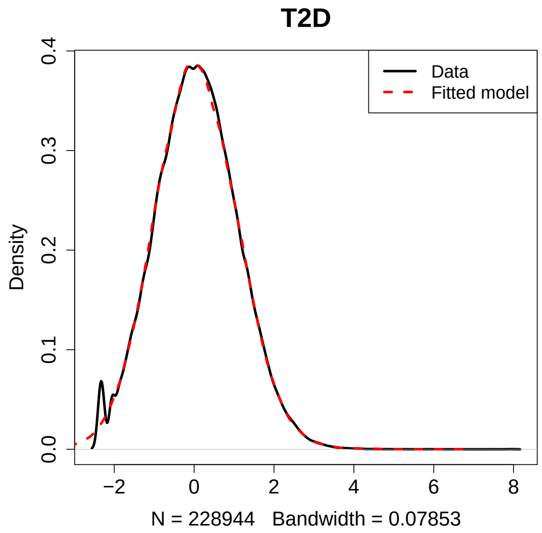

(b) T2D.

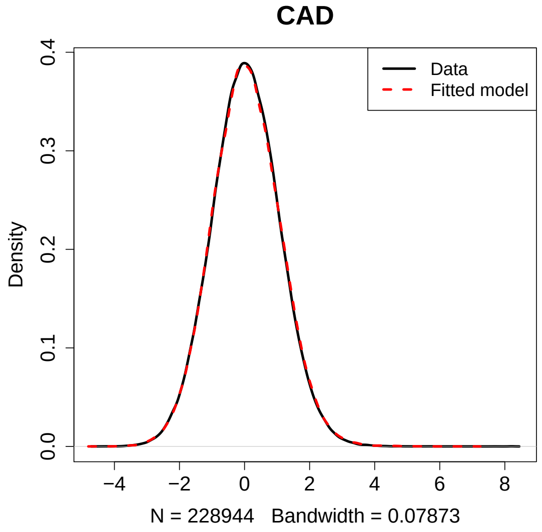

(c) CAD.

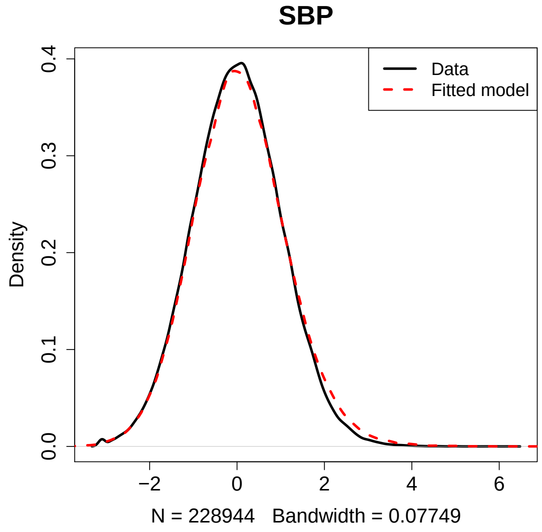

(d) SBP.

Figure J: Posterior predictive checking results for phenotypes HDL, T2D, CAD, and SBP.

8 GWAS of 12 phenotypes: Alternative emission distribution for associated SNPs

In order to evaluate robustness of the proposed graph-GPA model, we considered an alternative emission distribution for associated SNPs. Specifically, we replaced the distributional assumption of y_{it} in (1) of the main text with

$$p(y_{it}|e_{it}, a_y, b_{y_i}) = e_{it}\ \Gamma(y_{it}; a_y, b_{y_i}) + (1 - e_{it})\mathrm{N}(y_{it}; 0, 1),$$

where $\Gamma(y; a, b)$ denotes the gamma density with mean a/b evaluated at y. For convenience, we fixed $a_y = 2$ and put the conjugate prior distribution for b_{y_i} such that $b_{y_i} \sim \Gamma(\nu, \upsilon)$. We put $\nu = \upsilon = 1$ where the prior mean and variance of b_{y_i} are one. For posterior inference, we replace the Steps S1 − S3 of the original MCMC for the log-normal setting with the following steps:

S1. For each i and t, draw $e_{it} \sim \mathrm{Bernoulli}(p_1^*)$ where

$$p_1^* = \frac{\exp\left(\alpha_i + \sum_{j\sim i}\beta_{ij}e_{jt}\right)\cdot p(y_{it}|e_{it} = 1, a_y, b_{y_i})}{\sum_{e^*\in\{0,1\}}\exp\left(\alpha_i e^* + \sum_{j\sim i}\beta_{ij}\ e^*\ e_{jt}\right)\cdot p(y_{it}|e_{it} = e^*, a_y, b_{y_i})}.$$

S2. No update for a_y.

S3. For each i, draw b_{y_i} from its full conditional distribution,

$$b_{y_i} \sim \Gamma\left(\nu + n_i a_y, \quad \upsilon + \sum_{\{t: e_{it}=1\}} y_{it}\right)$$

where $n_i = \#\{t : e_{it} = 1\}$.

Table D shows the association mapping results for the case that we use Gamma density for the emission distribution for associated SNPs. The results are similar to the case that we use log-normal density for the emission distribution for associated SNPs and our conclusion essentially remains the same. Moreover, we found that using Gamma density for non-null distribution rather resulted in weaker sensitivity. Hence, we believe that this result justifies our choice of log-normal density for the emission distribution for associated SNPs.

Table D: GWAS of 12 phenotypes (graph-GPA analysis, when we use Gamma density for the emission distribution for associated SNPs): Numbers of SNPs identified to be associated with each pair of phenotypes by controlling the global FDR at nominal level of 10%. Diagonal elements show the number of SNPs inferred to be associated with each phenotype when the global FDR is controlled at the same level.

	ADHD	ASD	BPD	MDD	SCZ	RA	CD	UC	HDL	T2D	CAD	SBP
ADHD	0	0	0	0	0	0	0	0	0	0	0	0
ASD	0	6	0	0	0	1	0	0	0	0	0	0
BPD	0	0	32	0	8	1	1	0	0	0	0	0
MDD	0	0	0	8	0	0	0	0	0	0	0	0
SCZ	0	0	8	0	248	44	25	22	18	1	8	6
RA	0	1	1	0	44	442	173	150	14	12	6	9
CD	0	0	1	0	25	173	1258	466	53	9	21	5
UC	0	0	0	0	22	150	466	966	58	9	21	5
HDL	0	0	0	0	18	14	53	58	526	29	49	9
T2D	0	0	0	0	1	12	11	9	29	136	16	7
CAD	0	0	0	0	8	6	16	21	49	16	160	14
SBP	0	0	0	0	6	9	7	5	9	7	14	81

9 GWAS of 12 phenotypes: Convergence diagnostics

We check the convergence of MCMC run used in by trace plots. In Figure K, we can see that the MCMC chain quickly moves to a stationary marginal distribution with regard to six selected parameters: $|E|$, μ_1, σ_2^2, α_3, β_{34}, and β_{16}. The patterns of μ_i, σ_i^2 and α_i are similar for all the phenotypes i. The trace plot of β_{34} shows a typical pattern of trace plots for correlated pairs of phenotypes, while the pattern in β_{16} shows a typical pattern of trace plots for uncorrelated pairs of phenotypes. Note that we used the last 40,000 iterations in posterior inference by tossing out the first 10,000 iterations as burn-in.

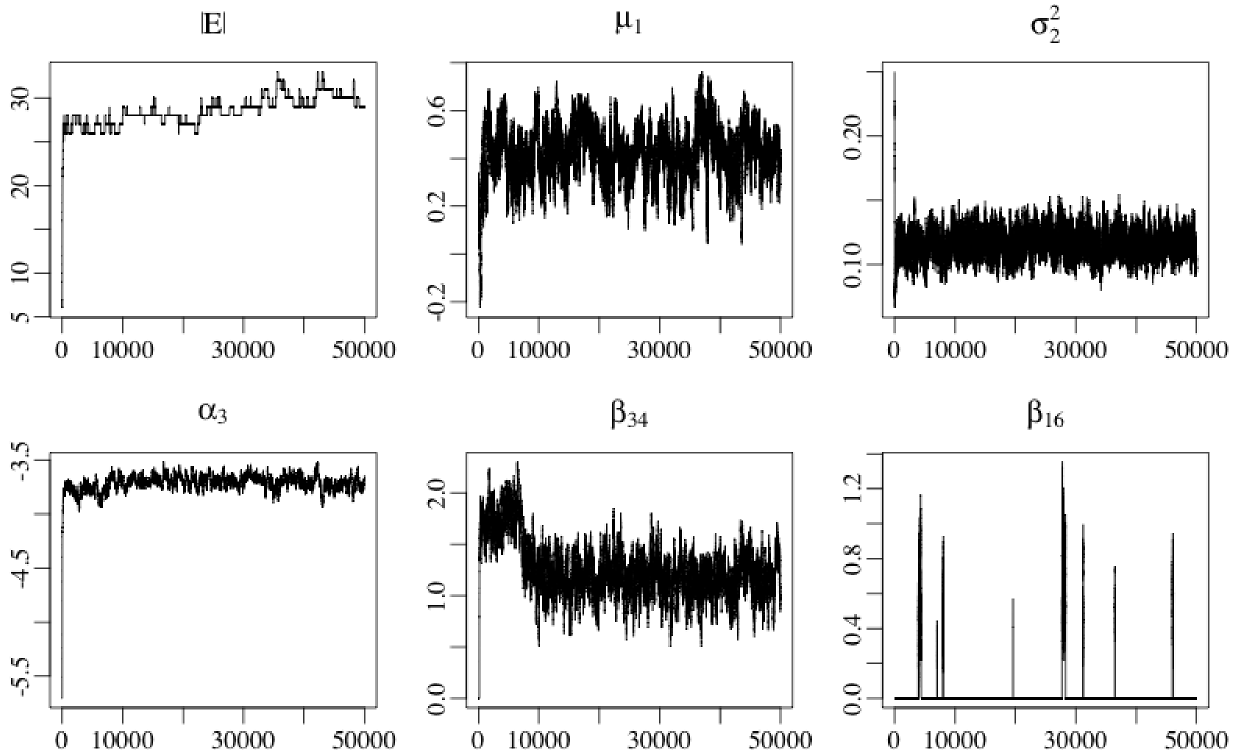

Figure K: Trace plots of six selected parameters for 50,000 iterations. The MCMC chain quickly moves to a stationary marginal distribution with regard to the parameters arbitrarily selected. The patterns of other parameters' trace plots are similar to those in this figures.

10 GWAS of 12 phenotypes: Model robustness to prior distributions

The proposed model is designed to use weakly informative prior distributions. In order to evaluate the robustness of the proposd model with respect to specification of prior distributions, we checked model sensitivity to a_σ, b_σ, a_β and b_β among the hyperparameters. For this purpose, we repeated the analysis in the main text by changing these hyperparameters, while all the other parameters were fixed as described in the main text.

Model A. Set $a_\beta = 0.5$ and $b_\beta = 0.5$, so that prior mean and variance of β_{ij} are 1 and 2.

Model B. Set $a_\sigma = 3$ and $b_\sigma = 6$, so that prior mean and variance of σ^2 are 3 and 9. Note that the sample ranges of logarithm-transformed values of $\boldsymbol{y}_i$ are from 4.1 to 9.4 for the 12 phenotypes and the sample standard deviations are around 1.05 for all phenotypes.

Table E: Numbers of SNPs identified from Model A by controlling the global FDR for phenotypes i and j at nominal level of 10%. Diagonal elements show the number of SNPs identified by controlling the global FDR for phenotype i at nominal level of 10%.

	ADHD	ASD	BPD	MDD	SCZ	RA	CD	UC	HDL	T2D	CAD	SBP
ADHD	0	0	0	0	0	0	0	0	0	0	0	0
ASD	0	8	0	0	0	0	0	0	0	0	0	0
BPD	0	0	79	0	32	7	13	2	3	0	0	0
MDD	0	0	0	19	0	0	0	0	0	0	0	0
SCZ	0	0	32	0	413	70	59	45	38	2	18	17
RA	0	0	7	0	70	686	291	262	38	11	16	17
CD	0	0	13	0	59	291	2336	846	133	23	39	21
UC	0	0	2	0	45	262	846	1786	111	14	34	12
HDL	0	0	3	0	38	38	133	111	890	66	96	15
T2D	0	0	0	0	2	11	23	14	66	275	56	12
CAD	0	0	0	0	18	16	39	34	96	56	318	47
SBP	0	0	0	0	17	17	21	12	15	12	47	168

Table F: Numbers of SNPs identified from Model B by controlling the global FDR for phenotypes i and j at nominal level of 10%. Diagonal elements show the number of SNPs identified by controlling the global FDR for phenotype i at nominal level of 10%.

	ADHD	ASD	BPD	MDD	SCZ	RA	CD	UC	HDL	T2D	CAD	SBP
ADHD	0	0	0	0	0	0	0	0	0	0	0	0
ASD	0	9	0	0	0	0	0	0	0	0	0	0
BPD	0	0	65	0	11	5	7	1	2	0	0	0
MDD	0	0	0	15	0	0	0	0	0	0	0	0
SCZ	0	0	11	0	410	70	47	41	39	0	17	14
RA	0	0	5	0	70	684	290	259	32	9	17	17
CD	0	0	7	0	47	290	2321	828	135	21	40	20
UC	0	0	1	0	41	259	828	1768	112	13	35	12
HDL	0	0	2	0	39	32	135	112	876	46	103	14
T2D	0	0	0	0	0	9	21	13	46	257	54	7
CAD	0	0	0	0	17	17	40	35	103	54	331	47
SBP	0	0	0	0	14	17	20	12	14	7	47	161

11 GWAS of 12 phenotypes: Graph estimation

Table G: GWAS of 12 phenotypes: Estimates of $p(E(i,j)|\boldsymbol{Y})$. The blanked cell indicates the zero estimated value.

	ADHD	ASD	BPD	MDD	SCZ	RA	CD	UC	HDL	T2D	CAD	SBP
ADHD	–	0.04	1.00	0.04	0.01	0.02	1.00	0.02	1.00			0.02
ASD	0.04	–	0.03			1.00	0.01			0.64	0.30	0.75
BPD	1.00	0.03	–	1.00	1.00	0.07	0.43		1.00		0.02	0.37
MDD	0.04		1.00	–	1.00	0.88			1.00			
SCZ	0.01		1.00	1.00	–	1.00	1.00		1.00			1.00
RA	0.02	1.00	0.07	0.88	1.00	–	1.00	1.00	1.00	0.01		0.01
CD	1.00	0.01	0.43		1.00	1.00	–	1.00	0.41	1.00	0.21	1.00
UC	0.02					1.00	1.00	–	1.00			
HDL	1.00		1.00	1.00	1.00	1.00	0.41	1.00	–	1.00	1.00	0.10
T2D		0.64				0.01	1.00		1.00	–	1.00	0.57
CAD		0.30	0.02				0.21		1.00	1.00	–	1.00
SBP	0.02	0.75	0.37		1.00	0.01	1.00		0.10	0.57	1.00	–

Table H: GWAS of 12 phenotypes: Posterior mean estimates of β_{ij}. The blanked cell indicates that $p(E(i,j)|\boldsymbol{Y})$ is estimated as zero and the bold number indicates that the 95% credible interval β_{ij} does not contain zero.

	ADHD	ASD	BPD	MDD	SCZ	RA	CD	UC	HDL	T2D	CAD	SBP
ADHD	–	0.02	**2.00**	0.04	0.00	0.01	**1.28**	0.01	**1.55**			0.01
ASD	0.02	–	0.02			**1.70**	0.01			0.53	0.30	1.07
BPD	**2.00**	0.02	–	**1.17**	**1.54**	0.04	0.17		**0.99**		0.01	0.27
MDD	0.04		**1.17**	–	**1.33**	0.80			**0.99**			
SCZ	0.00		**1.54**	**1.33**	–	**1.21**	**0.42**		**0.75**			**1.17**
RA	0.01	**1.70**	0.04	0.80	**1.21**	–	**1.90**	**1.44**	**1.20**	0.00		0.00
CD	**1.28**	0.01	0.17		**0.42**	**1.90**	–	**2.46**	0.29	**0.68**	0.10	**0.85**
UC	0.01					**1.44**	**2.46**	–	**1.14**			
HDL	**1.55**		**0.99**	**0.99**	**0.75**	**1.20**	0.29	**1.14**	–	**1.82**	**2.21**	0.06
T2D		0.53				0.00	**0.68**		**1.82**	–	**1.54**	0.46
CAD		0.30	0.01				0.10		**2.21**	**1.54**	–	**2.82**
SBP	0.01	1.07	0.27		**1.17**	0.00	**0.85**		0.06	0.46	**2.82**	–

12 GWAS of 12 phenotypes: graph-GPA analysis using RA subcohorts

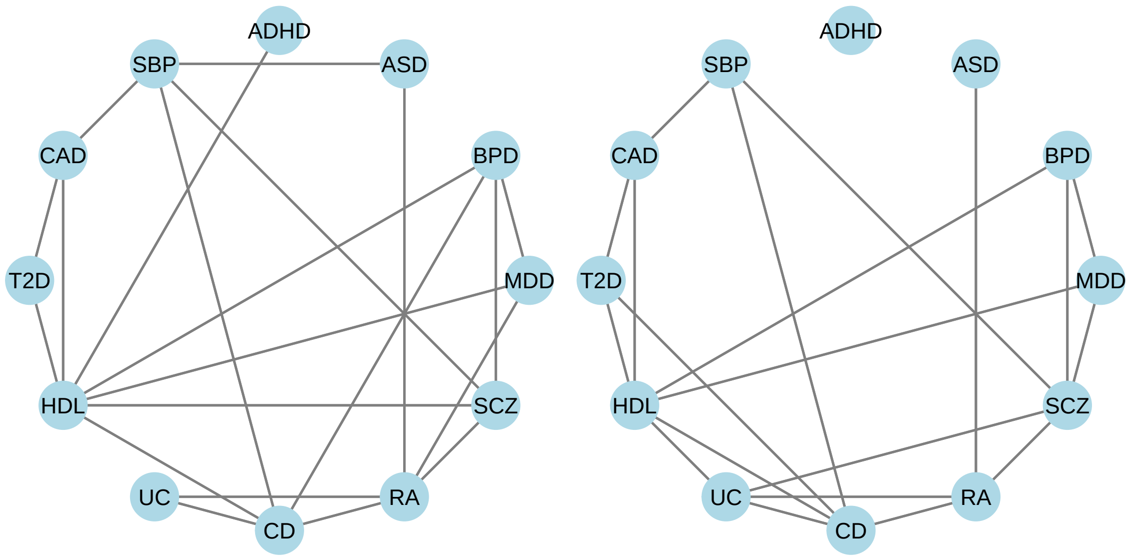

(a) Phenotype graph estimated using graph-GPA based on the GWAS data with the first RA cohort group.

(b) Phenotype graph estimated using graph-GPA based on the GWAS data with the second RA cohort group.

Figure L: GWAS of 12 phenotypes: Phenotype graph estimated using the graph-GPA model for GWAS data with two different RA cohort groups.

Table I: Numbers of SNPs identified from the graph-GPA model applied to the GWAS data with the first RA cohort group, by controlling the global FDR for phenotypes i and j at nominal level of 10%. Diagonal elements show the number of SNPs identified by controlling the global FDR for phenotype i at nominal level of 10%.

	ADHD	ASD	BPD	MDD	SCZ	RA	CD	UC	HDL	T2D	CAD	SBP
ADHD	0	0	0	0	0	0	0	0	0	0	0	0
ASD	0	10	0	0	0	0	0	0	0	0	0	0
BPD	0	0	89	0	39	4	15	0	4	0	0	0
MDD	0	0	0	21	0	1	0	0	0	0	0	0
SCZ	0	0	39	0	420	55	64	35	44	5	19	18
RA	0	0	4	1	55	444	216	170	10	9	12	15
CD	0	0	15	0	64	216	2294	796	129	18	38	21
UC	0	0	0	0	35	170	796	1695	79	18	36	13
HDL	0	0	4	0	44	10	129	79	869	67	97	16
T2D	0	0	0	0	5	9	18	18	67	277	59	14
CAD	0	0	0	0	19	12	38	36	97	59	317	46
SBP	0	0	0	0	18	15	21	13	16	14	46	172

Table J: Numbers of SNPs identified from the separate analyses with the GWAS data with the first RA cohort group, by controlling the global FDR for phenotypes i and j at nominal level of 10%. Diagonal elements show the number of SNPs identified by controlling the global FDR for phenotype i at nominal level of 10%.

	ADHD	ASD	BPD	MDD	SCZ	RA	CD	UC	HDL	T2D	CAD	SBP
ADHD	0	0	0	0	0	0	0	0	0	0	0	0
ASD	0	7	0	0	0	0	0	0	0	0	0	0
BPD	0	0	30	0	0	0	0	0	0	0	0	0
MDD	0	0	0	12	0	0	0	0	0	0	0	0
SCZ	0	0	0	0	271	1	2	0	0	0	0	0
RA	0	0	0	0	1	396	48	53	0	0	0	0
CD	0	0	0	0	2	48	1554	224	29	0	0	0
UC	0	0	0	0	0	53	224	1043	24	0	0	0
HDL	0	0	0	0	0	0	29	24	723	8	2	0
T2D	0	0	0	0	0	0	0	0	8	161	1	0
CAD	0	0	0	0	0	0	0	0	2	1	139	2
SBP	0	0	0	0	0	0	0	0	0	0	2	102

Table K: Numbers of SNPs identified from the graph-GPA model applied to the GWAS data with the second RA cohort group, by controlling the global FDR for phenotypes i and j at nominal level of 10%. Diagonal elements show the number of SNPs identified by controlling the global FDR for phenotype i at nominal level of 10%.

	ADHD	ASD	BPD	MDD	SCZ	RA	CD	UC	HDL	T2D	CAD	SBP
ADHD	0	0	0	0	0	0	0	0	0	0	0	0
ASD	0	11	0	0	0	0	0	0	0	0	0	0
BPD	0	0	73	0	23	2	8	2	2	0	0	0
MDD	0	0	0	16	0	0	0	0	0	0	0	0
SCZ	0	0	23	0	405	51	47	55	33	6	22	20
RA	0	0	2	0	51	320	159	157	11	16	15	14
CD	0	0	8	0	47	159	2246	800	124	30	41	23
UC	0	0	2	0	55	157	800	1717	101	19	36	15
HDL	0	0	2	0	33	11	124	101	866	69	99	16
T2D	0	0	0	0	6	16	30	19	69	283	63	14
CAD	0	0	0	0	22	15	41	36	99	63	323	51
SBP	0	0	0	0	20	14	23	15	16	14	51	172

Table L: Numbers of SNPs identified from the separate analyses with the GWAS data with the second RA cohort group, by controlling the global FDR for phenotypes i and j at nominal level of 10%. Diagonal elements show the number of SNPs identified by controlling the global FDR for phenotype i at nominal level of 10%.

	ADHD	ASD	BPD	MDD	SCZ	RA	CD	UC	HDL	T2D	CAD	SBP
ADHD	0	0	0	0	0	0	0	0	0	0	0	0
ASD	0	7	0	0	0	0	0	0	0	0	0	0
BPD	0	0	30	0	0	0	0	0	0	0	0	0
MDD	0	0	0	12	0	0	0	0	0	0	0	0
SCZ	0	0	0	0	271	0	2	0	0	0	0	0
RA	0	0	0	0	0	277	37	48	0	0	0	0
CD	0	0	0	0	2	37	1555	224	29	0	0	0
UC	0	0	0	0	0	48	224	1043	24	0	0	0
HDL	0	0	0	0	0	0	29	24	723	8	2	0
T2D	0	0	0	0	0	0	0	0	8	160	1	0
CAD	0	0	0	0	0	0	0	0	2	1	139	2
SBP	0	0	0	0	0	0	0	0	0	0	2	102

13 GWAS of 12 phenotypes: graph-GPA analysis with less stringent FDR controls

Table M: GWAS of 12 phenotypes (joint analysis using graph-GPA): Numbers of SNPs identified to be associated with each pair of phenotypes by controlling the global FDR at nominal level of 50%. Diagonal elements show the number of SNPs inferred to be associated with each phenotype when the global FDR is controlled at the same level.

	ADHD	ASD	BPD	MDD	SCZ	RA	CD	UC	HDL	T2D	CAD	SBP
ADHD	47	0	34	7	29	14	39	23	38	0	0	0
ASD	0	182	0	0	20	79	49	37	15	20	25	22
BPD	34	0	1595	173	609	148	286	218	298	68	93	68
MDD	7	0	173	416	260	125	143	112	162	32	55	37
SCZ	29	20	609	260	5652	445	761	577	478	152	250	215
RA	14	79	148	125	445	1985	1190	1043	318	139	119	69
CD	39	49	286	143	761	1190	18263	7560	816	481	408	269
UC	23	37	218	112	577	1043	7560	15830	822	345	328	143
HDL	38	15	298	162	478	318	816	822	3186	698	765	189
T2D	0	20	68	32	152	139	481	345	698	2573	620	200
CAD	0	25	93	55	250	119	408	328	765	620	2489	510
SBP	0	22	68	37	215	69	269	143	189	200	510	1210

Table N: GWAS of 12 phenotypes (separate analysis): Numbers of SNPs identified to be associated with each pair of phenotypes by controlling the global FDR at nominal level of 50%. Diagonal elements show the number of SNPs inferred to be associated with each phenotype when the global FDR is controlled at the same level.

	ADHD	ASD	BPD	MDD	SCZ	RA	CD	UC	HDL	T2D	CAD	SBP
ADHD	0	0	0	0	0	0	0	0	0	0	0	0
ASD	0	71	0	0	0	0	0	0	0	0	0	0
BPD	0	0	594	0	9	0	2	5	0	1	0	0
MDD	0	0	0	85	0	0	0	0	0	0	0	0
SCZ	0	0	9	0	4298	73	75	82	39	1	20	11
RA	0	0	0	0	73	1512	262	271	15	16	0	9
CD	0	0	2	0	75	262	14046	998	153	37	22	17
UC	0	0	5	0	82	271	998	11066	124	18	26	2
HDL	0	0	0	0	39	15	153	124	2293	45	52	8
T2D	0	0	1	0	1	16	37	18	45	1525	20	3
CAD	0	0	0	0	20	0	22	26	52	20	1271	13
SBP	0	0	0	0	11	9	17	2	8	3	13	831

14 GWAS of 12 phenotypes: GPA analysis

Table O: GWAS of 12 phenotypes (joint analysis using GPA): Numbers of SNPs identified to be associated with each pair of phenotypes by controlling the global FDR at nominal level of 10%. Diagonal elements show the number of SNPs inferred to be associated with each phenotype when the global FDR is controlled at the same level. Note that for the diagonal elements, we show the number of associated SNPs averaged over the pairs estimated to be correlated with each phenotype.

	ADHD	ASD	BPD	MDD	SCZ	RA	CD	UC	HDL	T2D	CAD	SBP
ADHD	0	0	0	0	0	0	0	0	0	0	0	0
ASD	0	563	0	0	0	539	0	0	0	0	0	0
BPD	0	0	707	0	401	93	0	0	691	0	0	32
MDD	0	0	0	68	63	157	0	0	0	0	0	0
SCZ	0	0	401	63	640	545	16	18	71	0	9	25
RA	0	539	93	157	545	570	576	576	10	22	1	15
CD	0	0	0	0	16	576	2194	3037	131	12	8	6
UC	0	0	0	0	18	576	3037	1763	135	3	9	0
HDL	0	0	691	0	71	10	131	135	798	165	166	19
T2D	0	0	0	0	0	22	12	3	165	302	43	4
CAD	0	0	0	0	9	1	8	9	166	43	295	56
SBP	0	0	32	0	25	15	6	0	19	4	56	281

15 GWAS of 12 phenotypes: GenoCanyon and GenoSkyline annotation of the graph-GPA analysis results

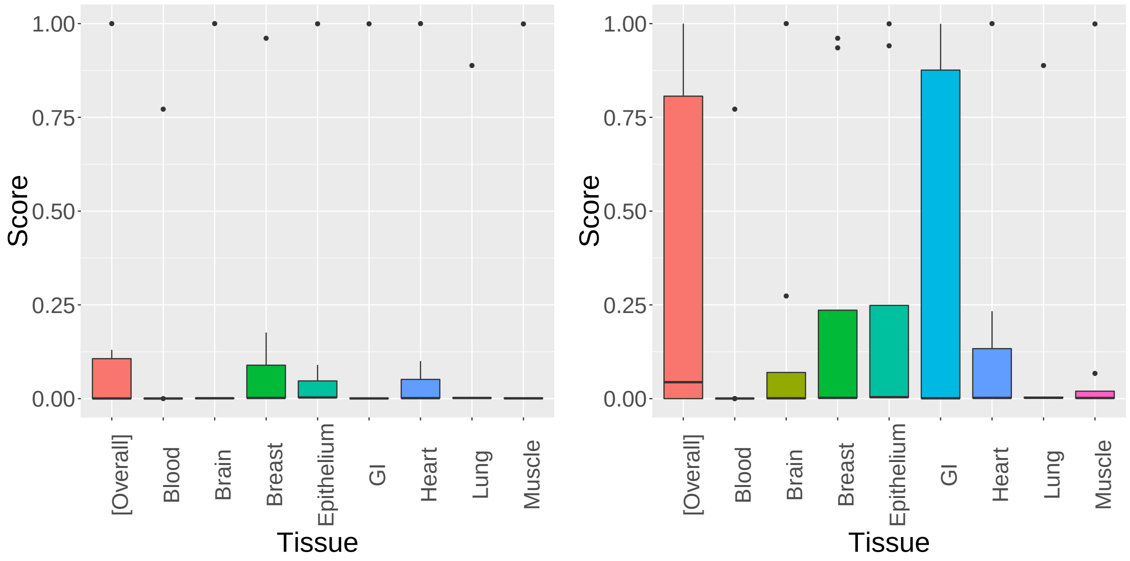

(a) Functional importance: Separate analysis.

(b) Functional importance: Joint analysis using graph-GPA.

Figure M: GeneCanyon ([overall]) and GeneSkyline scores for various tissues for the SNPs associated with autism spectrum disorder (ASD) in a separate analysis (a) and a joint analysis using graph-GPA (b).

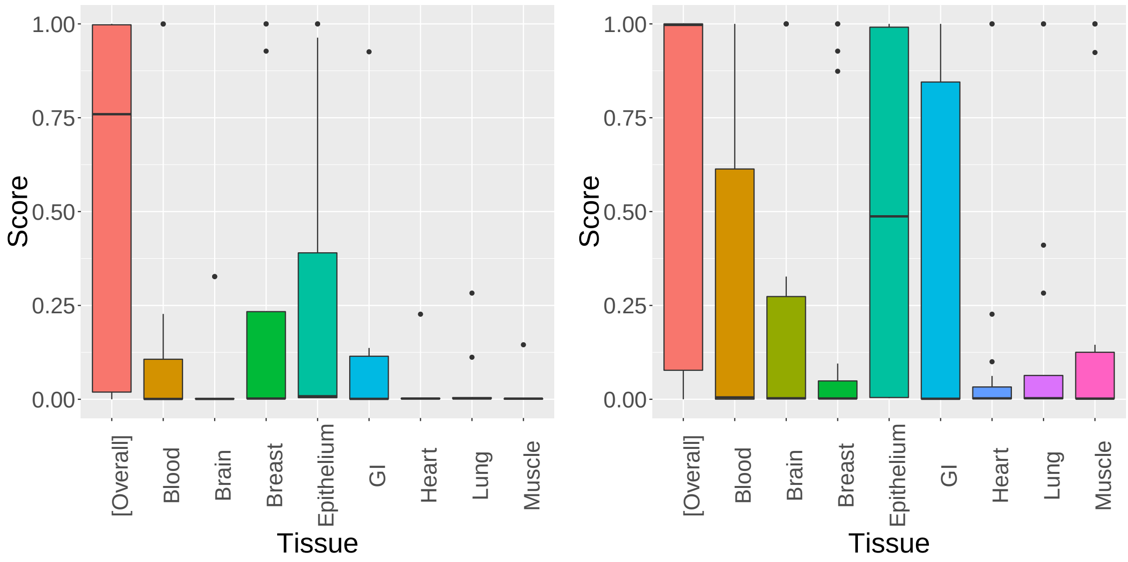

(a) Functional importance: Separate analysis.

(b) Functional importance: Joint analysis using graph-GPA.

Figure N: GeneCanyon ([overall]) and GeneSkyline scores for various tissues for the SNPs associated with major depression disorder (MDD) in a separate analysis (a) and a joint analysis using graph-GPA (b).

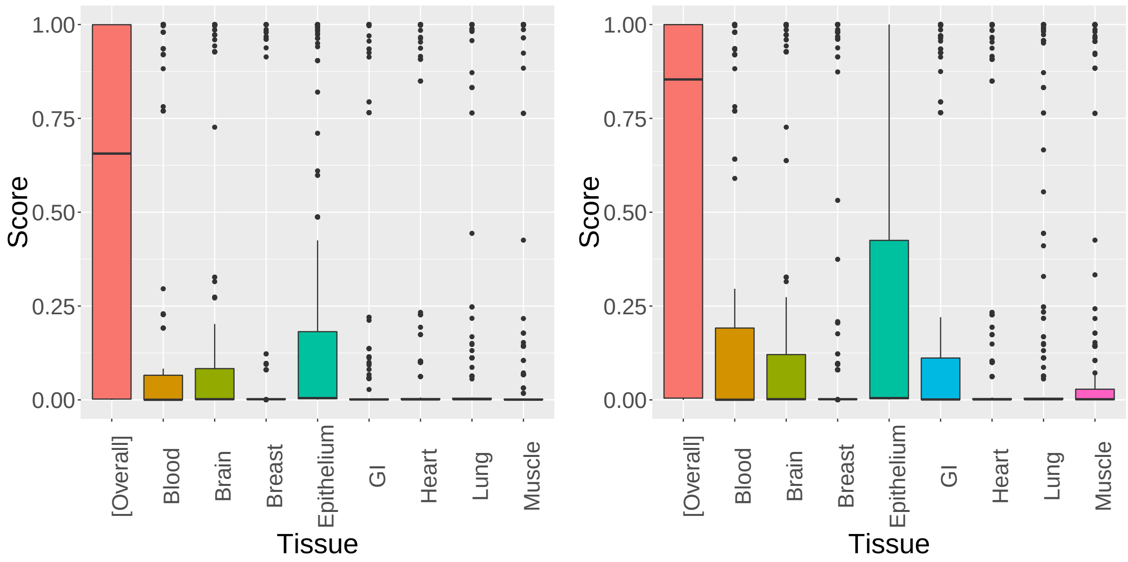

(a) Functional importance: Separate analysis.

(b) Functional importance: Joint analysis using graph-GPA.

Figure O: GeneCanyon ([overall]) and GeneSkyline scores for various tissues for the SNPs associated with schizophrenia (SCZ) in a separate analysis (a) and a joint analysis using graph-GPA (b).

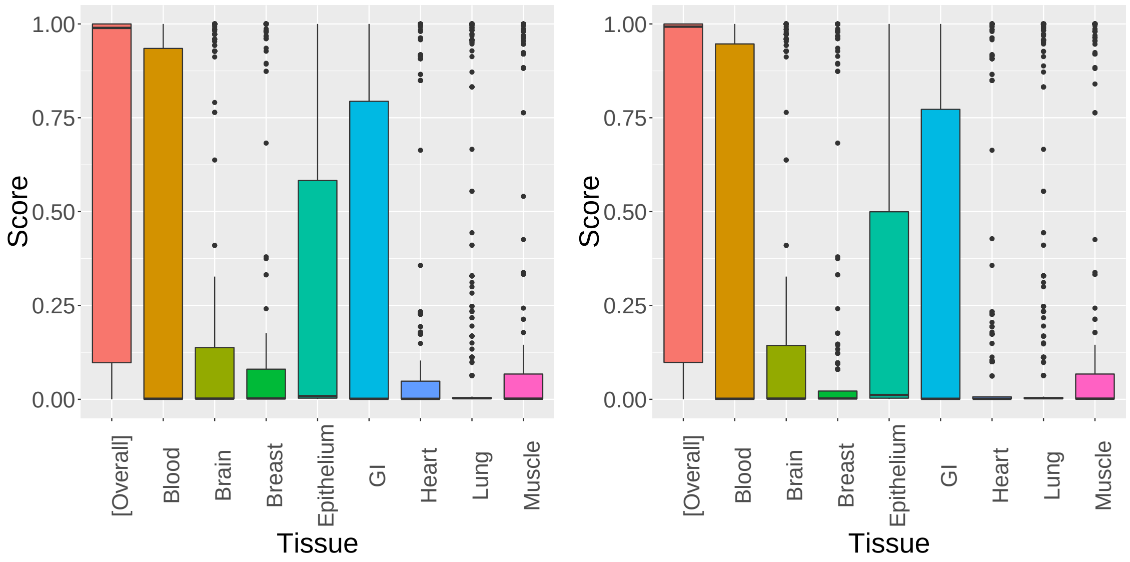

(a) Functional importance: Separate analysis.

(b) Functional importance: Joint analysis using graph-GPA.

Figure P: GeneCanyon ([overall]) and GeneSkyline scores for various tissues for the SNPs associated with rheumatoid arthritis (RA) in a separate analysis (a) and a joint analysis using graph-GPA (b).

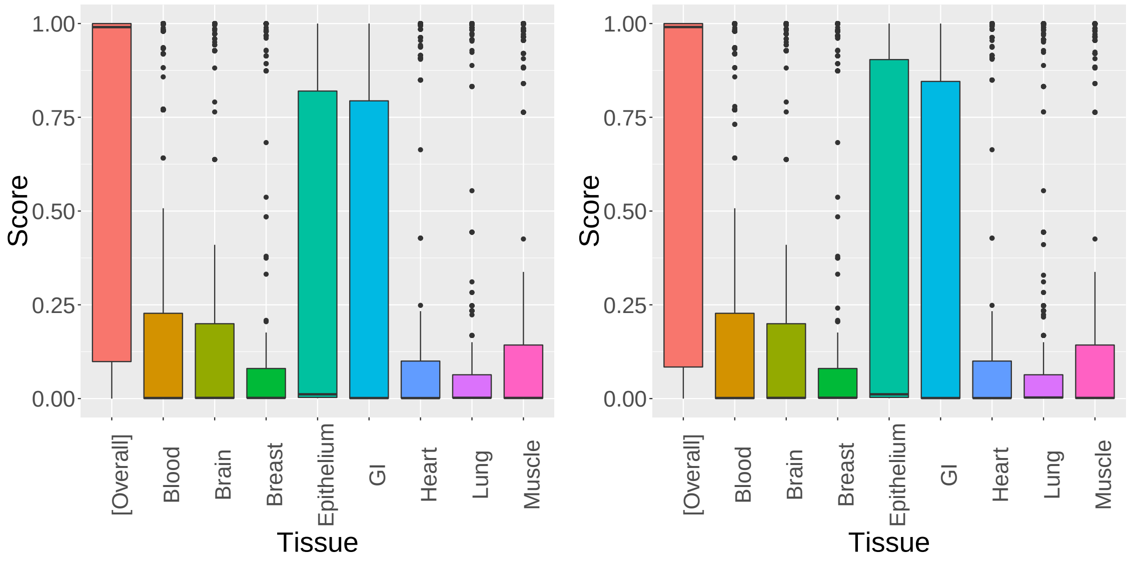

(a) Functional importance: Separate analysis.

(b) Functional importance: Joint analysis using graph-GPA.

Figure Q: GeneCanyon ([overall]) and GeneSkyline scores for various tissues for the SNPs associated with high-density lipoprotein (HDL) in a separate analysis (a) and a joint analysis using graph-GPA (b).

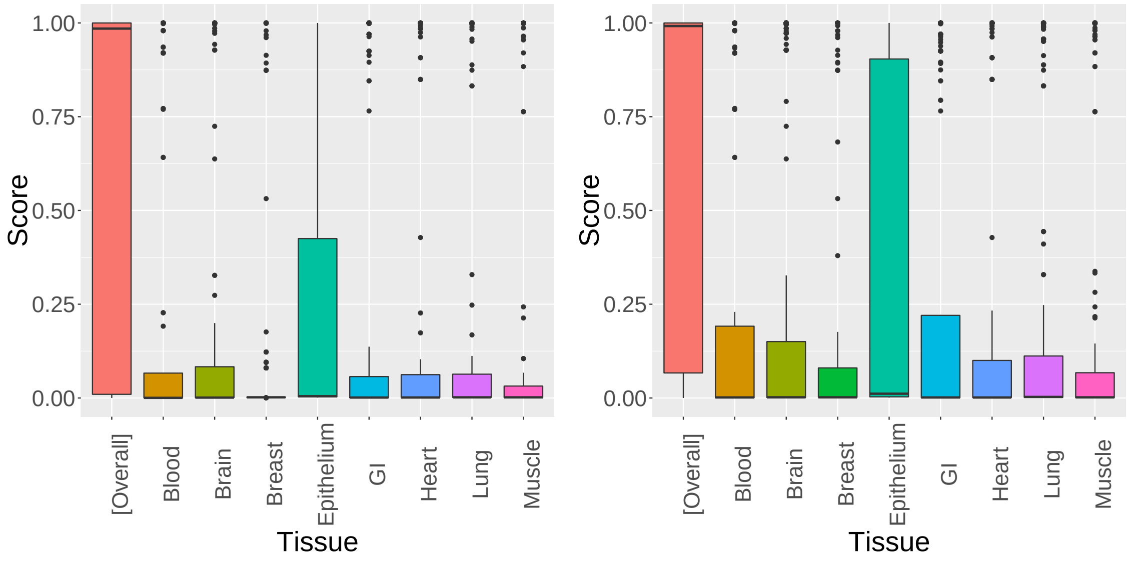

(a) Functional importance: Separate analysis.

(b) Functional importance: Joint analysis using graph-GPA.

Figure R: GeneCanyon ([overall]) and GeneSkyline scores for various tissues for the SNPs associated with type 2 diabetes (T2D) in a separate analysis (a) and a joint analysis using graph-GPA (b).

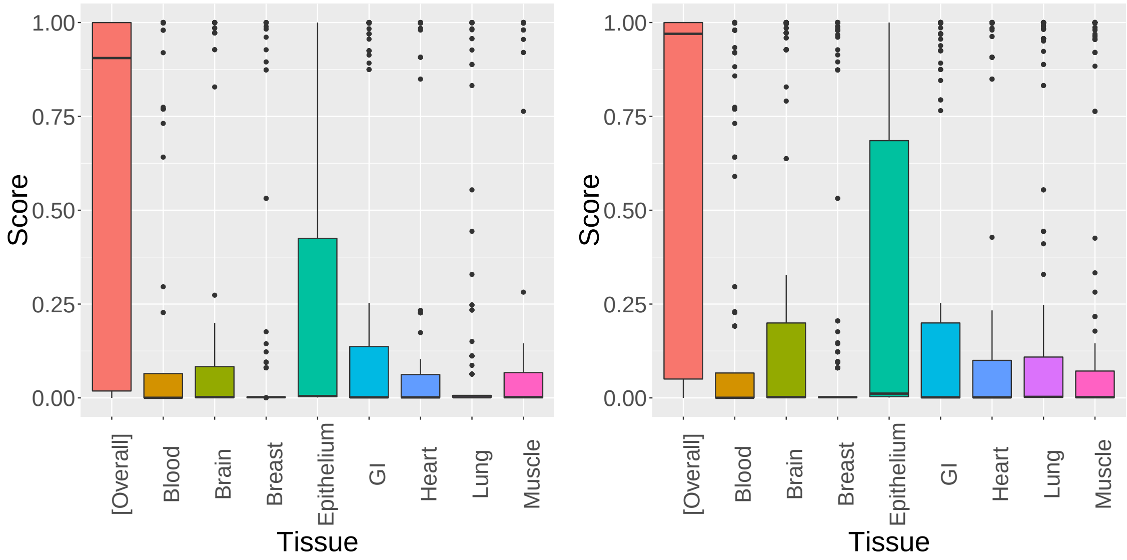

(a) Functional importance: Separate analysis.

(b) Functional importance: Joint analysis using graph-GPA.

Figure S: GeneCanyon ([overall]) and GeneSkyline scores for various tissues for the SNPs associated with coronary artery disease (CAD) in a separate analysis (a) and a joint analysis using graph-GPA (b).

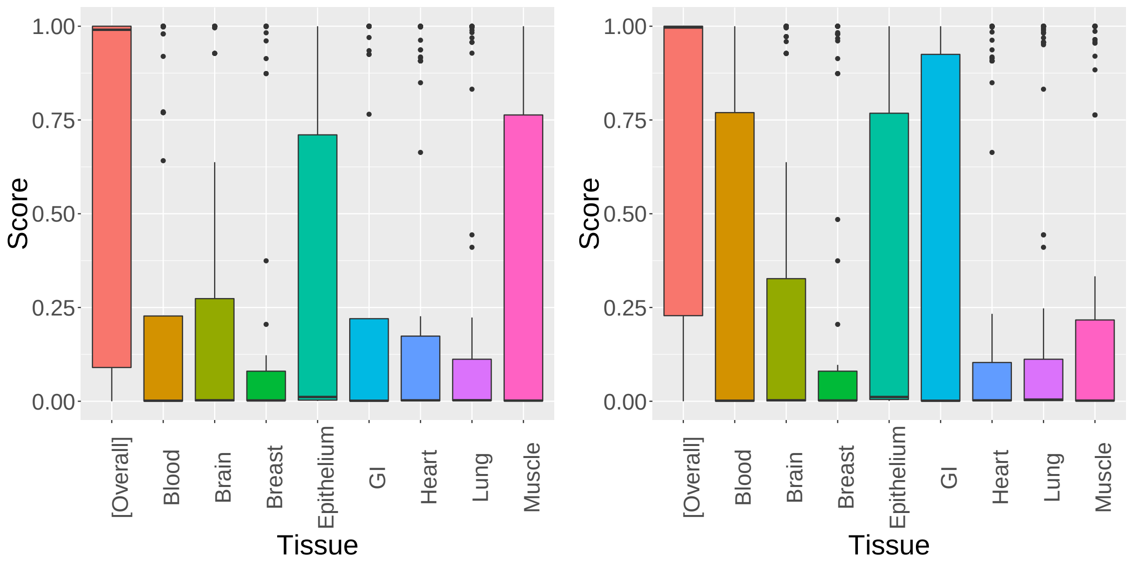

(a) Functional importance: Separate analysis.

(b) Functional importance: Joint analysis using graph-GPA.

Figure T: GeneCanyon ([overall]) and GeneSkyline scores for various tissues for the SNPs associated with systolic blood pressure (SBP) in a separate analysis (a) and a joint analysis using graph-GPA (b).

16 GWAS of Bipolar Disorder: GenoSkyline annotation of the graph-GPA analysis results for brain tissue

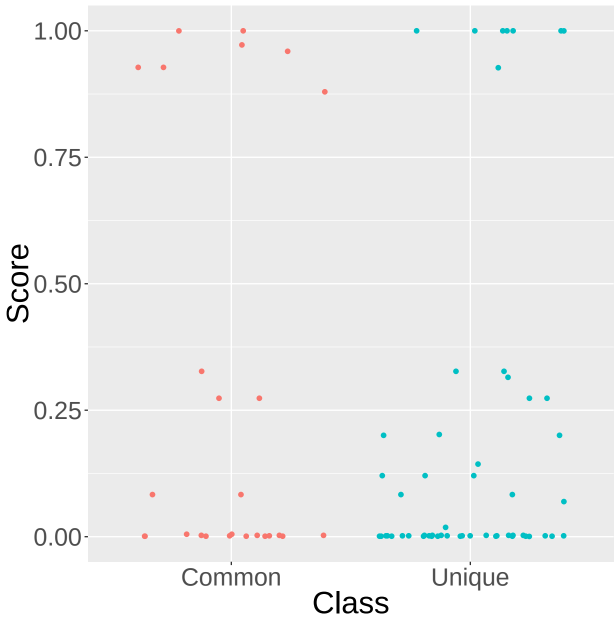

Figure U: GeneSkyline scores for brain tissue for the SNPs associated with bipolar disorder (BPD). The points of light blue color ('Unique') indicates the SNPs identified only by the graph-GPA analysis, and the points of pink color ('Common') indicates the SNPs identified in both separate and graph-GPA analyses.

17 Impact of overlapping subjects on the estimation of pleiotropic architecture

We investigated the impact of overlapping subjects on the estimation of pleiotropic architecture by extending our simulation studies. Specifically, we consider the same pleiotropic structure ($\mathbf{G}$) and the same set of associated SNPs ($\mathbf{E}$) that were assumed in our simulation studies, where the first five phenotypes ($P1 - P5$) are genetically correlated and there are two independent phenotypes (P6 and P7). Then, given the specified pleiotropic architecture ($\mathbf{G}$) and the specified genotype-phenotype association status ($\mathbf{E}$), we generated p-values using the classical liability threshold model. Specifically, the minor allele frequencies (MAF) of 20,000 SNPs were drawn from $U[0.05, 0.5]$ and the per-minor-allele effect of each risk SNP was drawn from $N(0, h^2/(1-h^2)f_j(1-f_j)m)$, where h^2 is the desired level of variance explained by all SNPs on the liability scale, f_j is the MAF of the corresponding j-th SNP, and m is the number of associated SNPs. We also simulated the environmental effect on the liability scale for each individual from $N(0, 1)$. The total liability for each individual was then obtained by adding up all the genetic effects and the environmental effect. Given a desired disease prevalence B, individuals with liabilities greater than the $1 - B$ quantile were classified as cases and others were classified as controls. Then equal numbers of cases and controls were drawn from the cohort as a GWAS data set. We assumed that $h^2 = 0.6$, $B = 0.1$, 5,000 cases, and 5,000 controls. Finally, we obtained the p-value for each SNP in each disease using a χ^2-test with one degree of freedom.

In order to mimic the overlapping subject situation, we considered various proportion of controls shared between P6 and P7 (γ). Because P6 and P7 are designed to be independent, the estimated correlation between P6 and P7 can be considered as a pure artifact. Moreover, because $P1 - P5$ are designed to be genetically correlated, we can evaluate the impact of shared subjects on the estimated pleiotropic architecture by comparing confidence about the edges between P6 and P7 with confidence about the edges among $P1 - P5$. Tables $P - T$ show the association mapping results for $\gamma = 0, 0.25, 0.5, 0.75, 1$, where $\gamma = 0$ corresponds to no overlapping subjects and $\gamma = 1$ means that all controls are shared. We can see that sharing of subjects up to $\gamma = 0.75$ (75% of controls are shared; Table S) essentially does not generate artificial correlation between P6 and P7 in the sense that no edge was identified between P6 and P7 in the estimated phenotype graph and no SNP was identified to be shared between these two phenotypes. We observed some artificial correlation between P6 and P7 when all controls are shared ($\gamma = 1$; Table T). Specifically, in this case, an edge between P6 and P7 was identified and 19 SNPs were called to be shared between these two phenotypes. However, we note that we still identified a much smaller number of SNPs shared between P6 and P7, compared to those shared among $P1 - P5$ ($28 - 164$ SNPs were identified to be shared between these pairs). Moreover, when we take into account numbers of SNPs associated with each phenotype, the proportion of SNPs shared between P6 and P7 was still significantly

smaller than numbers of SNPs shared among P1 − P5. For example, 28 SNPs were identified to be shared between P3 and P4 while 194 and 411 SNPs were determined to be associated with each of P3 and P4, respectively. In contrast, only 19 SNPs were identified to be shared between P6 and P7, although 463 and 351 SNPs were determined to be associated with each of P6 and P7, respectively. In summary, although the proposed graph-GPA model does not explicitly take into account the issue of overlapping subjects, its estimation of pleiotropic architecture is still robust to overlapping subjects. Moreover, even when this artificial phenotypic correlation is generated, the confidence assigned to this correlation is still significantly lower than that assigned to the pairs of phenotype that are truly correlated.

Table P: Numbers of SNPs identified to be associated with each pair of phenotypes for the overlapping subject situation simulation with $\gamma = 0$. The global FDR at nominal level of 10% is used. Diagonal elements show the number of SNPs inferred to be associated with each phenotype when the global FDR is controlled at the same level. The bold number indicates that the phenotypes are correlated, i.e., $p(E(i,j)|\boldsymbol{Y}) > 0.5$ and $p(\beta_{ij} > 0|\boldsymbol{Y}) > 0.95$.

	P1	P2	P3	P4	P5	P6	P7
P1	370	**122**	**61**	19	7	5	0
P2	**122**	491	**53**	15	9	1	0
P3	**61**	**53**	194	**28**	13	1	0
P4	19	15	**28**	411	**164**	6	0
P5	7	9	13	**164**	472	9	4
P6	5	1	1	6	9	437	0
P7	0	0	0	0	4	0	293

Table Q: Numbers of SNPs identified to be associated with each pair of phenotypes for the overlapping subject situation simulation with $\gamma = 0.25$. The global FDR at nominal level of 10% is used. Diagonal elements show the number of SNPs inferred to be associated with each phenotype when the global FDR is controlled at the same level. The bold number indicates that the phenotypes are correlated, i.e., $p(E(i,j)|\boldsymbol{Y}) > 0.5$ and $p(\beta_{ij} > 0|\boldsymbol{Y}) > 0.95$.

	P1	P2	P3	P4	P5	P6	P7
P1	370	**122**	**60**	19	7	7	0
P2	**122**	491	**53**	15	9	0	1
P3	**60**	**53**	194	**28**	13	0	0
P4	19	15	**28**	411	**164**	5	1
P5	7	9	13	**164**	471	7	4
P6	7	0	0	5	7	423	0
P7	0	1	0	1	4	0	297

Table R: Numbers of SNPs identified to be associated with each pair of phenotypes for the overlapping subject situation simulation with $\gamma = 0.5$. The global FDR at nominal level of 10% is used. Diagonal elements show the number of SNPs inferred to be associated with each phenotype when the global FDR is controlled at the same level. The bold number indicates that the phenotypes are correlated, i.e., $p(E(i,j)|\boldsymbol{Y}) > 0.5$ and $p(\beta_{ij} > 0|\boldsymbol{Y}) > 0.95$.

	P1	P2	P3	P4	P5	P6	P7
P1	370	**122**	**61**	19	7	4	0
P2	**122**	490	**53**	15	9	0	0
P3	**61**	**53**	194	**28**	13	0	0
P4	19	15	**28**	411	**164**	3	1
P5	7	9	13	**164**	471	6	6
P6	4	0	0	3	6	414	0
P7	0	0	0	1	6	0	290

Table S: Numbers of SNPs identified to be associated with each pair of phenotypes for the overlapping subject situation simulation with $\gamma = 0.75$. The global FDR at nominal level of 10% is used. Diagonal elements show the number of SNPs inferred to be associated with each phenotype when the global FDR is controlled at the same level. The bold number indicates that the phenotypes are correlated, i.e., $p(E(i,j)|\boldsymbol{Y}) > 0.5$ and $p(\beta_{ij} > 0|\boldsymbol{Y}) > 0.95$.

	P1	P2	P3	P4	P5	P6	P7
P1	370	**122**	**61**	19	7	5	0
P2	**122**	491	**53**	15	9	0	0
P3	**61**	**53**	194	**28**	13	0	0
P4	19	15	**28**	411	**164**	6	1
P5	7	9	13	**164**	471	8	5
P6	5	0	0	6	8	445	0
P7	0	0	0	1	5	0	291

Table T: Numbers of SNPs identified to be associated with each pair of phenotypes for the overlapping subject situation simulation with $\gamma = 1.0$. The global FDR at nominal level of 10% is used. Diagonal elements show the number of SNPs inferred to be associated with each phenotype when the global FDR is controlled at the same level. The bold number indicates that the phenotypes are correlated, i.e., $p(E(i,j)|\boldsymbol{Y}) > 0.5$ and $p(\beta_{ij} > 0|\boldsymbol{Y}) > 0.95$.

	P1	P2	P3	P4	P5	P6	P7
P1	370	**122**	**61**	19	7	5	0
P2	**122**	491	**53**	15	9	1	0
P3	**61**	**53**	194	**28**	13	0	0
P4	19	15	**28**	411	**164**	7	2
P5	7	9	13	**164**	471	9	5
P6	5	1	0	7	9	463	**19**
P7	0	0	0	2	5	**19**	351
